# DNA-encoded nucleosome occupancy is associated with transcription levels in the human malaria parasite Plasmodium falciparum

**DOI:** 10.1186/1471-2164-15-347

**Published:** 2014-05-08

**Authors:** Evelien M Bunnik, Anton Polishko, Jacques Prudhomme, Nadia Ponts, Sarjeet S Gill, Stefano Lonardi, Karine G Le Roch

**Affiliations:** Department of Cell Biology and Neuroscience, Center for Disease Vector Research, Institute for Integrative Genome Biology, University of California, Riverside, 900 University Avenue, Riverside, CA 92521 USA; Department of Computer Science and Engineering, University of California, Riverside, Riverside, CA 92521 USA; Mycology and Food Safety, INRA Centre de Bordeaux-Aquitaine, Villenave d’Ornon Cedex, 33883 France

**Keywords:** Malaria, Cell cycle, Nucleosome, Transcription, Sequence

## Abstract

**Background:**

In eukaryotic organisms, packaging of DNA into nucleosomes controls gene expression by regulating access of the promoter to transcription factors. The human malaria parasite *Plasmodium falciparum* encodes relatively few transcription factors, while extensive nucleosome remodeling occurs during its replicative cycle in red blood cells. These observations point towards an important role of the nucleosome landscape in regulating gene expression. However, the relation between nucleosome positioning and transcriptional activity has thus far not been explored in detail in the parasite.

**Results:**

Here, we analyzed nucleosome positioning in the asexual and sexual stages of the parasite’s erythrocytic cycle using chromatin immunoprecipitation of MNase-digested chromatin, followed by next-generation sequencing. We observed a relatively open chromatin structure at the trophozoite and gametocyte stages, consistent with high levels of transcriptional activity in these stages. Nucleosome occupancy of genes and promoter regions were subsequently compared to steady-state mRNA expression levels. Transcript abundance showed a strong inverse correlation with nucleosome occupancy levels in promoter regions. In addition, AT-repeat sequences were strongly unfavorable for nucleosome binding in *P. falciparum*, and were overrepresented in promoters of highly expressed genes.

**Conclusions:**

The connection between chromatin structure and gene expression in *P. falciparum* shares similarities with other eukaryotes. However, the remarkable nucleosome dynamics during the erythrocytic stages and the absence of a large variety of transcription factors may indicate that nucleosome binding and remodeling are critical regulators of transcript levels. Moreover, the strong dependency between chromatin structure and DNA sequence suggests that the *P. falciparum* genome may have been shaped by nucleosome binding preferences. Nucleosome remodeling mechanisms in this deadly parasite could thus provide potent novel anti-malarial targets.

**Electronic supplementary material:**

The online version of this article (doi:10.1186/1471-2164-15-347) contains supplementary material, which is available to authorized users.

## Background

The genomes of eukaryotic organisms are wrapped around histone proteins to form a condensed protein-DNA complex known as chromatin [1]. The basic packaging unit, the nucleosome, consists of approximately 147 base pairs of DNA wrapped around a histone octamer, containing two copies of each of the histone proteins H2A, H2B, H3, and H4. The positioning of these nucleosomes along the genome is important for control of gene expression by regulating access of DNA to transcription factors and other DNA binding proteins [2–5]. Nucleosomes are therefore not arranged randomly, but show a distinct distribution around genes. This nucleosome landscape was first observed in *Saccharomyces cerevisiae*, and was later confirmed for higher eukaryotes, such as worms, flies, and humans (comprehensively reviewed in [6, 7]). In summary, the core promoter is characterized by a nucleosome-depleted region followed by a strongly positioned +1 nucleosome that overlaps the transcription start site. The coding region is packaged by an array of nucleosomes with increasingly fuzzy positioning towards the 3′ end. Finally, another nucleosome-depleted region is present at the 3′ end of the gene where RNA polymerase II terminates transcription.

Nucleosome positioning is influenced by binding of other proteins and protein complexes to the DNA that act as barriers, as well as by chromatin remodeling enzymes that actively shape the nucleosome landscape. However, one of the strongest determinants of nucleosome positioning is the DNA sequence itself. In eukaryotes such as *S. cerevisiae* and humans, relatively rigid stretches of deoxyadenosines (poly[dA:dT]) decrease nucleosome binding affinity [8, 9]. Poly(dA:dT) tracts are often found in the nucleosome-depleted regions that demarcate eukaryotic core promoters [2, 3, 5] and their length and location relative to the transcription start site (TSS) have been shown to influence promoter activity [10]. In addition, nucleosome-bound DNA shows a 10 bp periodicity of anti-phased A/T and G/C dinucleotides that corresponds to the helical turn of DNA wrapped around the histone core [11–13].

The human malaria parasite, *Plasmodium falciparum*, yearly responsible for an estimated 660,000 deaths [14], has the most highly AT-rich genome sequenced to date. The AT-content averages 80.7% genome-wide, but reaches 90-95% in intergenic regions [15]. Similar to other eukaryotic genomes, the *P. falciparum* genome is bound by nucleosomes, but the formation of tightly packed heterochromatin may be prevented by the absence of linker histone H1, of which to date no homologue has been detected in *P. falciparum*. The *P. falciparum* genome is relatively depleted of transcription factors, but encodes a large amount of chromatin remodeling enzymes [16]. Accordingly, the nucleosome landscape of *P. falciparum* has been proposed to be important for regulation of gene expression during the stage of its life cycle responsible for disease in humans [17–19]. This asexual erythrocytic stage is characterized by parasite invasion of a red blood cell, followed by multiplication and the release of 16-32 daughter cells after a cell cycle of approximately 48 hours. Differentiation of asexual parasites into male or female gametocytes allows transmission of *P. falciparum* to mosquitos. Understanding the regulatory mechanisms for sexual differentiation is critical for the development of strategies aimed at preventing transmission of malaria.

In a previous study, we analyzed the nucleosome landscape of the *P. falciparum* genome during the asexual cycle by a combination of micrococcal nuclease (MNase) assisted isolation of nucleosome-bound DNA (MAINE) and formaldehyde-assisted isolation of protein-free DNA (FAIRE), coupled to next-generation sequencing [18]. Due to the relatively low coverage of these data sets (generated in 2008), we were unable to perform an in-depth study of correlations between transcriptional activity and nucleosome positioning, in particular for promoter regions. Here, we generated a new, high-coverage, nucleosome positioning data set and uncovered a strong dependency between sequence composition, nucleosome occupancy and transcriptional activity of both promoter and gene regions during the asexual and sexual stages of *P. falciparum*. This study confirms that one of the main determinants of nucleosome positioning in eukaryotes is encoded in the DNA sequence itself and that changes in nucleosome occupancy greatly influence gene expression. However, given the strong dynamics in nucleosome positioning observed during the erythrocytic cycle and the relative paucity of transcription factors, these results suggest that the parasite may have developed its nucleosome landscape as a key mechanism to regulate gene expression.

## Results

### Generation of high-resolution nucleosome positioning maps

A detailed analysis of the relation between the nucleosome landscape and gene expression during the human malaria parasite’s asexual cycle has thus far been hampered by a lack of high-resolution nucleosome maps. In addition, nucleosome maps were not yet available for the sexually mature gametocyte stage. To extend and complement existing nucleosome occupancy data sets [17, 18, 20], we performed two variants of chromatin immunoprecipitation coupled to next generation sequencing (ChIP-Seq) for nucleosome mapping using either sonication (sonication ChIP-Seq) or MNase digestion (MNase ChIP-Seq) to fragment crosslinked chromatin into mononucleosomes (Additional file 1: Figure S1). Antibodies used for immunoprecipitations were either directed against H4, which is universally present in all nucleosomes, or against an epitope shared between H3 and H3.3, which enabled pull-down of all nucleosomes with the exception of centromeric nucleosomes that contain the variant histone CenH3. Nucleosome-bound DNA fragments were subsequently analyzed by next-generation sequencing (Illumina HiSeq 2000), generating 50 base pair (bp) paired-end sequence reads. Reads were first mapped to the human genome, followed by mapping of all remaining reads to the *P. falciparum* genome (Additional file 1: Figure S1, Table S1).

While digestion of chromatin by MNase has been reported to be biased by the preference of MNase for AT-rich sequences [21], others have found no evidence for such claim [22]. The use of a non-enzymatic fragmentation method and the specific enrichment for nucleosome-bound DNA by immunoprecipitation should rule out that nucleosome-sized fragments were obtained merely as a potential sequence-based artifact of MNase digestion. MNase digestion resulted in a narrow distribution of mononucleosomal DNA fragments (average size = 130 bp) while sonication yielded larger fragments (average size = 253 bp; Additional file 1: Figure S2). Sonication thus seems to leave relatively long tails of DNA that are not part of the nucleosome itself, and may sometimes yield intact di- or trinucleosomes, indicating that MNase-based fragmentation of chromatin provides a higher resolution as compared to sonication.

**Figure 1:**
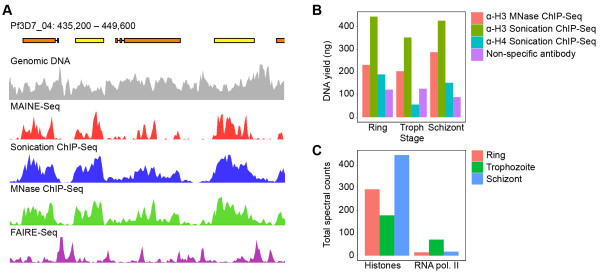
**Validation of data sets. A**. Genome browser view (Artemis [56]) showing genome coverage for a section of chromosome 4, with the gene model indicated in the top. Genes located on the forward and the reverse strands are indicated in yellow and orange, respectively. Sequencing of genomic DNA resulted in an even distribution of reads across intergenic and coding regions. Sequence read coverage for MAINE-Seq [18], Sonication ChIP-Seq and MNase ChIP-Seq was similar and showed increased coverage of coding region as compared to intergenic regions, while opposite results were observed for FAIRE-Seq [18]. **B**. DNA yields of immunoprecipitations using a non-specific antibody, an antibody directed against histone H3 (Abcam ab1971) and an antibody directed against histone H4 (Millipore 05-858). These data show a reduced amount of DNA obtained from trophozoite samples, despite using the same amount of input material for each immunoprecipitation. Since low amounts of DNA were obtained using the anti-H4 antibody, we concluded that this antibody was not well suited for ChIP-Seq experiments and was therefore not used for further analysis. **C**. Total spectral counts for all histone proteins (left) and all annotated RNA polymerase II proteins (right) obtained by mass spectrometry analysis of nuclear fractions from ring, trophozoite and schizont stage parasites [24]. Abundance levels of histones decreased during the trophozoite stage, while the amount of RNA polymerase II increased, consistent with high transcriptional activity in this stage, but not the ring or schizont stages. Spectral count data were obtained from PlasmoDB (http://www.plasmodb.org).

Extensive visual inspection of MAINE-Seq [18] and sonication/MNase ChIP-Seq tracks in a genome browser confirmed very similar distribution patterns across the genome (Figure 1A). Accordingly, we observed high correlations between MAINE-Seq [18], MNase ChIP-Seq, and sonication ChIP-Seq (Additional file 1: Figure S3), indicating that we obtained similar nucleosome maps despite differences in methodology. We therefore used MNase ChIP-Seq data for all subsequent analyses of the asexual cycle. However, as ChIP-Seq generally yields relatively low amounts of DNA, we analyzed the nucleosome landscape of sexually mature parasites (gametocytes) using MAINE-Seq.

### Transcriptionally active stages show global depletion in nucleosome levels

Nucleosome dynamics were analyzed in synchronized *P. falciparum* cultures that were harvested directly after invasion of red blood cells at the ring stage, and at the trophozoite and schizont stages, as well as at the sexually mature gametocyte stage. The amount of chromatin used for immunoprecipitations was equivalent for all stages of the asexual cell cycle. However, DNA yields obtained from ChIPs consistently showed a reduced recovery at the trophozoite stage (Figure 1B). These results are consistent with data from a wide variety of experimental approaches, including western blot analysis [23] and mass spectrometry analysis [24] (Figure 1C), that showed reduced histone levels at the trophozoite stage, which is known to be the most transcriptionally active stage during the asexual cell cycle [25, 26]. While detection of histone proteins decreased at the trophozoite stage as compared to the ring and schizont stages, mass spectrometry data showed an increased abundance of RNA polymerase II [24] (Figure 1C), further validating a potential link between histone levels and transcriptional activity.

Next, we determined the number of nucleosomes and the average nucleosome level at each stage using the nucleosome positioning tool PuFFIN [27]. PuFFIN was designed in our group specifically for paired-end sequence reads. Extensive experimental results showed that PuFFIN outperforms other available nucleosome detection tools in terms of accuracy and number of nucleosomes reported along the genome. The number of reads mapped to each nucleosome was used to estimate nucleosome levels. To compare nucleosome levels between different stages, we normalized nucleosome scores using the total number of reads mapped to both human and *P. falciparum* genomes (see Materials and methods). In addition, we validated our nucleosome positioning data using a publicly available nucleosome positioning tool NOrMAL [28].

**Figure 2:**
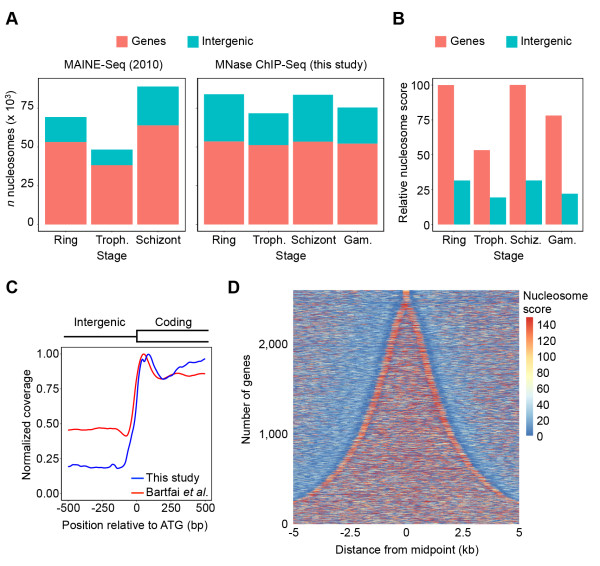
**Nucleosome positioning analysis across the erythrocytic cell cycle. A**. The total number of nucleosomes in intergenic and coding regions as detected in our old MAINE-Seq data set (left panel) [18] compared to the total number of nucleosomes reported by PuFFIN using our new high-coverage MNase ChIP-Seq data set (right panel). **B**. Average scores of nucleosomes located in intergenic and coding regions at different time points during the asexual and sexual stages of the erythrocytic cell cycle. Values are expressed as the percentage of the average score of nucleosomes located inside genes at the ring stage. **C**. Average genome-wide sequence read coverage around the translation start site for a previously published nucleosome occupancy data set [20]) and the MNase ChIP-Seq data set from this study. Both data sets show a well-positioned first nucleosome inside the coding region, as well as reduced coverage in intergenic regions as compared to coding regions. Coverage is expressed as the fraction of the highest coverage value among all positions within the plot window. **D**. Nucleosomal organization in and around coding regions. The location and scores of nucleosomes detected by PuFFIN are shown for all 2,262 *P. falciparum* genes that do not contain any introns. Each line represents one gene, sorted from small (top) to large (bottom) and centered around its midpoint. More strongly positioned nucleosomes are represented by increasingly warmer colors. Troph.: trophozoite; schiz.: schizont; gam.: gametocyte.

The number of detected nucleosomes substantially increased in comparison with previous low-coverage MAINE-Seq data [18] (Figure 2A). The number of locations where nucleosomes were present did not change dramatically during the cell cycle (Figure 2A) and were not greatly affected by differences in sequencing library size between the different stages (Additional file 1: Figure S4). However, nucleosome scores showed approximately a two-fold drop at the trophozoite stage, both inside genes and in intergenic regions (Figure 2B), similar to reductions in ChIP DNA yield (Figure 1B) and histone abundance at this stage (Figure 1C). In agreement with a previous data set [18], these results suggest a binary structure of the chromatin, with strong nucleosome packaging early in the cell cycle, nucleosome depletion in trophozoite stage to allow massive transcription and DNA replication, and tight re-compaction at the late stage in preparation for egress of daughter cells and re-invasion of new red blood cells. Furthermore, nucleosome positioning obtained from sexually mature gametocytes also showed a relatively open chromatin structure, consistent with the level of transcriptional activity at this stage (Figure 2).

Nucleosome occupancy inside genes was higher as compared to the relatively nucleosome-depleted intergenic regions (Figure 2B). Reduced sequence read coverage in intergenic regions as compared to coding regions was also observed for a previously published nucleosome occupancy data set [20], albeit to a lower extent (Figure 2C). The most strongly positioned nucleosomes were observed at the start and the end of coding regions (Figure 2D). Between these two strongly positioned nucleosomes, nucleosomal organization in *P. falciparum* genes appears to be relatively random (Figure 2D), in contrast to the evenly spaced arrays of nucleosomes observed inside genes of other eukaryotes. To validate that the low coverage observed in intergenic regions was not mainly due to an artifact introduced by the low recovery of sequencing AT-rich regions, we sequenced parasite genomic DNA, which resulted in an even coverage distribution over intergenic and coding regions (Figure 1A). In addition, sequencing results from non-protein-bound DNA (FAIRE-Seq) [18] showed a high enrichment of intergenic regions, with more than 50% of all sequence reads mapped to intergenic regions (Additional file 1: Figure S5). These data suggest that the reduced intergenic coverage in our nucleosome-bound DNA sequencing data set is not simply the result of technical difficulties in sequencing the highly AT-rich intergenic regions of the *P. falciparum* genome. Similar nucleosome maps were obtained using sonication ChIP-Seq, although the number of mapped nucleosomes was lower due to the lower resolution of this data set (Additional file 1: Figure S6). Collectively, these results confirm that dramatic changes in nucleosome landscape occur during the transcriptionally most active erythrocytic stages of *P. falciparum*.

### Nucleosome occupancy in promoter regions strongly correlates with transcription levels

**Figure 3:**
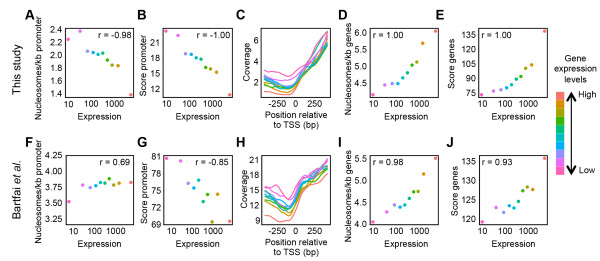
**Association between nucleosome occupancy and transcription level at the trophozoite stage. A**. The average number of nucleosomes per kilobase in the 500 base pair upstream of the translation start site (promoter region) for each transcription cluster of 500 genes. **B**. The average score of nucleosomes located in the promoter region for each transcription cluster. **C**. Sequence read coverage around the transcription start site for the subset of genes with annotated transcription start sites in each transcription cluster. **D**. The average number of nucleosomes per kilobase inside coding regions for each transcription cluster. **E**. The average score of all nucleosomes located inside coding regions for each transcription cluster. Correlation coefficients reported were obtained using the Spearman’s rank test. **F-J**: Same as panels **A-E** for an independent trophozoite-stage nucleosome occupancy data set generated by Bartfai *et al*. [20].

In other eukaryotic organisms, decreased nucleosome occupancy in promoter regions is associated with higher transcriptional activity [4]. For *P. falciparum*, this connection has not been studied in detail. Our most recent high coverage nucleosome and transcriptome [29] data sets, both obtained at the exact same parasitic stages, allowed us to analyze the impact of nucleosome occupancy in promoter regions on gene expression in *P. falciparum*. We computed nucleosome occupancy levels for the 500 base pair region directly upstream of the translation start codon at the trophozoite stage. Genes were grouped into ten transcription clusters of 500 genes each, based on steady-state mRNA levels. We observed a strong correlation between nucleosome density in the promoter region (both in terms of number of nucleosomes and nucleosome level) and transcriptional activity, with highly expressed gene clusters showing a more open nucleosome organization than clusters of genes with low expression values (Figure 3A, B). In addition, for a smaller group of genes with annotated transcription start sites, promoter regions of highly expressed genes showed decreased nucleosome occupancy (Figure 3C). Inside coding regions of highly expressed genes, we observed an opposite correlation between nucleosome occupancy and transcriptional activity as compared to the promoter regions. Highly transcribed genes were on average bound by more nucleosomes and at higher levels (Figure 3D, E). A similar, albeit slightly weaker, association between nucleosome occupancy and transcript levels was observed when nucleosome maps were generated using the nucleosome positioning tool NOrMAL [28] (Additional file 1: Figure S7), indicating that this result is not an artifact of the PuFFIN algorithm. In addition, the more open chromatin structure at promoters of highly expressed genes was replicated in sonication ChIP-Seq data obtained using either anti-H3 or anti-H4 antibodies (Additional file 1: Figure S8), suggesting that this observation is independent of ChIP-Seq methodology.

To further validate our findings, we analyzed a previously published nucleosome occupancy data set generated using different experimental procedures for nucleosome isolation and library preparation [20]. We observed a similar correlation between gene expression level and nucleosome occupancy in both the promoter region and the coding region (Figure 3F-J), indicating that these findings are not a result of biases of the methodologies used.

Similar results were obtained using other publicly available RNA-Seq data sets [20, 26, 30] (Additional file 1: Figure S9). The correlation between nucleosome occupancy and transcription level was also observed when genes were divided into 100 clusters of 50 genes (Additional file 1: Figure S10), but was absent for randomly generated gene clusters (Additional file 1: Figure S11). To further investigate the dependency between nucleosome occupancy and transcript levels, we analyzed previously published transcriptome and nucleosome positioning data sets from yeast [31, 32] and human cells [33] using our analysis pipeline. In yeast, we observed a comparable, but weaker association between expression levels and nucleosome occupancy in the promoter region (Additional file 1: Figure S12). In human, the nucleosome landscape at the promoter region has a bimodal distribution, with a relatively open organization for expressed genes and a relatively closed organization for silent genes (Additional file 1: Figure S12). Transcriptional control in human cells is known to include more complex regulatory elements, such as enhancer regions that are located further away from the transcription start site. These results could therefore indicate that nucleosomal organization of promoter regions may be more important for regulation of transcriptional activity in organisms with compact genomes, such as *P. falciparum*, than in higher eukaryotes.

### Nucleosome occupancy patterns associated with stage-specific gene expression

**Figure 4:**
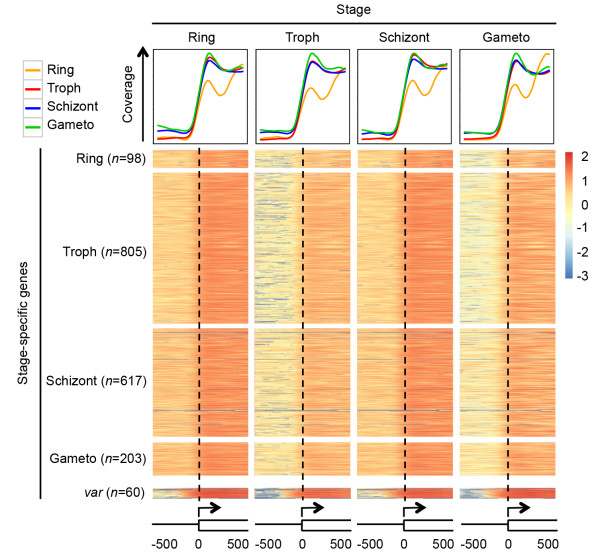
**Nucleosome occupancy in genes and promoter regions of stage-specifically expressed genes.** Coverage plots (top row) show average read coverage around the translation start site (-/+500 bp) for all genes with stage-specific expression profiles (as color-indicated in the top left corner) at the four different stages. The number of genes in each group is indicated on the left. At all time points, genes specifically expressed at the schizont and gametocyte (Gameto) stages show a slightly more closed chromatin organization in the promoter region. A relatively large proportion of ring stage-specific genes has a small first exon, resulting in a lower occupancy of the first nucleosome inside the coding region. Heatmap presents normalized read coverage per nucleotide for all genes within each group. Read coverage of the *var* genes is shown in the bottom row. Read coverage values are expressed on a log 10 scale. Troph: trophozoite stage.

Similar associations between transcriptional activity and nucleosome characteristics of the promoter region and gene region were observed at the ring and schizont stages of the asexual cycle, and at the gametocyte stage (Additional file 1: Figure S13). This may be the result of large overlaps between gene clusters with high transcription levels at different stages, as a large proportion of genes has high transcriptional activity throughout the erythrocytic cell cycle. A comparison of nucleosome occupancy for genes with stage-specific transcription profiles showed that changes in nucleosome occupancy occur at a genome-wide level (Figure 4). However, promoter regions of genes transcribed at the ring and trophozoite stage on average tend to be the most nucleosome-depleted, while promoter regions of genes expressed at the schizont and gametocyte stage have on average a slightly more compact nucleosome organization (Figure 4). Stage-specific transcription factors may be critical for regulating expression of these particular genes. In addition, we found that *var* genes, associated with antigenic variation and pathogenesis, are strongly bound by nucleosomes, while their promoters are relatively nucleosome-depleted (Figure 4). Transcription of the *var* gene family is known to be tightly regulated and involves mutually exclusive expression of one *var* gene out of a total of approximately 60 gene variants. Transcriptional repression of the remaining variants is achieved by a combination of upstream and intronic regulatory elements, nuclear localization in repressive centers and repressive histone marks [34–37]. The nucleosome landscape of this gene family is markedly different from non-*var* genes and could contribute to these alternative mechanisms of transcriptional regulation [17].

### AT-repeats are the strongest inhibitory sequences for nucleosome binding in P. falciparum

In other eukaryotes, DNA sequence has been shown to be an important determinant of nucleosome positioning. Poly(dA:dT) tracts, in particular, are frequently present in nucleosome-depleted promoter regions and inhibit nucleosome binding by decreasing the ability of the DNA helix to wrap around the nucleosome core [2, 3, 5][8–10].

**Figure 5:**
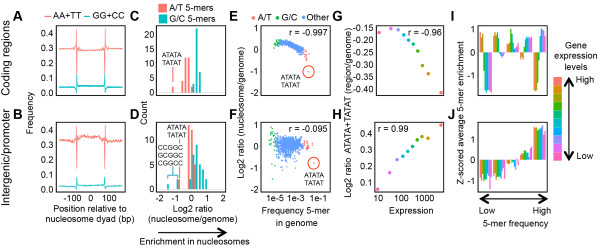
**Sequence-based nucleosome-binding preferences in** ***P. falciparum*** **. A-B**. Frequency of AA + TT and GG + CC dinucleotides in nucleosome-bound DNA fragments in coding regions **(A)** and intergenic regions **(B)**. For nucleosomes located inside genes, a weak 10 bp periodicity was observed directly internal to the nucleosome boundaries, but disappearing closer to the nucleosome dyad. **C-D**. Enrichment of 5-mers consisting exclusively of A/T or G/C nucleotides for nucleosomes in coding regions **(C)** and intergenic regions **(D)** at the ring stage. Enrichment is expressed as the log 2 ratio of the frequency of a 5-mer inside nucleosomes and the genome-wide frequency of that 5-mer. **E-F**. Genome-wide frequency of each 5-mer plotted against the enrichment of each 5-mer in nucleosomes, shown for nucleosomes in coding regions **(E)** and intergenic regions **(F)**. Nucleosomes inside coding regions show a strong binding preference for 5-mers with low genome-wide frequencies, while this association is absent for intergenic nucleosomes. AT-repeat 5-mers are indicated by a red circle. Pearson correlation coefficients are shown in the top right corner of each plot. **G-H**. Enrichment of AT-repeat 5-mers (ATATA and TATAT) in coding regions **(G)** and in the 500 nucleotides upstream of the translation start site (promoter region) **(H)** relative to their genome-wide frequency, for genes in each transcription cluster. Correlation coefficients were obtained using the Spearman’s rank test. **I-J**. Sequence composition in coding regions **(I)** and promoter regions **(J)** among genes with different expression levels. 5-mers were divided into five groups based on their genome-wide frequency in intergenic regions. For each transcription cluster, the z-normalized average log 2 ratio of 5-mer frequency in the gene or promoter versus all genes or intergenic regions, respectively, is plotted for all 5-mer frequency groups. Highly expressed genes show an increased prevalence of nucleosome-favorable 5-mers, while genes with low transcription levels show an increased prevalence of nucleosome-disfavorable 5-mers.

To investigate whether sequence-dependent nucleosome binding preferences are present in the AT-rich *P. falciparum* genome, we computed the average frequency of dinucleotides in all sequence reads that exactly matched the length of DNA wrapped around a nucleosome (i.e. 147 bp). In contrast to the strong 10 bp periodicity observed in other eukaryotes, we observed a weak 10 bp periodic signal for AA and TT dinucleotides in *P. falciparum* nucleosomes located inside genes, and no periodic signal in intergenic regions (Figure 5A, B). We also computed the enrichment of all 5-mers located in nucleosomes relative to the whole genome. In coding regions, nucleosome-bound DNA showed an enrichment of 5-mers exclusively composed of G/C and a depletion of 5-mers exclusively composed of A/T (Figure 5C), comparable to observations in yeast [38]. In contrast, A/T 5-mers were not as strongly depleted from nucleosomes located in intergenic regions, with the exception of 5-mers consisting of AT-repeats (ATATA and TATAT; Figure 5D). In addition, 5-mers containing a GG dinucleotide bordered by cytosines showed depletion from intergenic nucleosomes (CGGCC, CCGGC, and GCGGC; Figure 5D). Similar results were obtained using 4-mer or 6-mer distribution patterns (Additional file 1: Figure S14). Interestingly, intergenic nucleosomes showed a small enrichment for 5 bp poly(dA:dT) tracts, which were mainly found close to the nucleosome dyad (Additional file 1: Figure S15), while this 5-mer is strongly unfavorable for nucleosome binding in other eukaryotes. For nucleosomes located in genes, the level of 5-mer enrichment was inversely correlated with their genome-wide frequency (Figure 5E). This was not observed for intergenic nucleosomes, with the exception of 5-mers consisting of AT-repeats (Figure 5F).

Similar to poly(dA:dT) tracts in other eukaryotes, AT-repeats seem to be the most unfavorable sequences for nucleosomes in both gene and intergenic regions. Since AT-repeats are also the most abundant 5-mers in the *P. falciparum* genome, comprising 3.4% and 10.7% of gene and intergenic genome sequence, respectively, these sequences may have a profound effect on the nucleosome landscape of this parasite. Indeed, AT-repeat sequences were strongly depleted inside coding regions of highly expressed genes (Figure 5G, Additional file 1: Figure S16) and were enriched in promoters of highly expressed genes (Figure 5H, Additional file 1: Figure S16), corresponding to levels of nucleosome occupancy in these regions. Highly expressed genes showed a strong enrichment of 5-mers with low genome-wide frequency (Figure 5I, Additional file 1: Figure S16), while promoter regions of high and low expression genes did not show a difference in 5-mer composition (Figure 5J, Additional file 1: Figure S16).

## Discussion

Here, we studied nucleosome positioning in the human malaria parasite, and its association with transcription levels and DNA sequence. Our results suggest that nucleosome levels are partially DNA-encoded and constitute an important control mechanism of gene expression levels in *P. falciparum*. The association between DNA composition (mainly AT-repeat sequences), nucleosome landscape, and transcription levels observed in this study is similar to the effect of poly(dA:dT) tracts on nucleosome positioning and promoter activity in yeast [10, 38]. However, to the best of our knowledge, this is the first report that details their relation in *P. falciparum* and provides a genome-wide, *in vivo* validation of the original observations in yeast. In addition, this study exposes several distinct differences in nucleosome organization and dynamics between *P. falciparum* and other eukaryotes that will be discussed in more detail below.

### Nucleosome binding preferences

High AT-content, in particular in the form of poly(dA:dT) tracts, are known to influence nucleosome binding affinities [2, 3, 5][8–10]. However, the nucleosome binding preferences that we observed in *P. falciparum* are distinct from eukaryotes with lower genomic AT-contents. The typical 10 bp periodicity of A/T dinucleotides was absent, similar to observations in the AT-rich (77%) amoebe *Dictyostelium discoideum*[39]. In addition, AT-repeat sequences, not poly(dA:dT) tracts, seem to be a strong determinant of nucleosome positioning in *P. falciparum*. The *P. falciparum* histone H3 shows 92% homology to human H3 and 87% to *S. cerevisiae* H3, which could account for differences in nucleosome binding preferences. Furthermore, nucleosomes located in the intergenic regions of the *P. falciparum* genome have been found to contain histone variants H2A.Z and H2B.Z [40, 41]. The presence of these histone variants may promote nucleosome deposition in highly AT-rich regions, and could explain differences in binding preferences between genic and intergenic nucleosomes.

Given the high prevalence of AT-repeat sequences in the *P. falciparum* genome, it is tempting to speculate that the development of chromatin organization as a regulatory mechanism for gene expression levels has been the driving force for the evolution of the *P. falciparum* genome towards its high AT-content. This in turn raises the question how nucleosome positioning and transcriptional activity are connected in *Plasmodium* species with lower AT-content, such as *P. vivax* (57.7% AT [42]). However, since presently *P. vivax* cannot be maintained in a continuous culture, this question might be difficult to answer.

### Nucleosome landscape in and around coding regions

Nucleosomal organization around coding regions is relatively conserved across divergent eukaryotic species, with strongly positioned nucleosomes bordering nucleosome-depleted promoter regions [6, 7]. Downstream nucleosomes are arranged in tightly spaced arrays with increasing fuzziness towards the 3′ end of coding regions. In *P. falciparum*, we observed the most strongly positioned nucleosomes at the start and the end of coding regions, with less tightly organized nucleosomes in the gene body. The highly AT-rich intergenic regions may act as barriers determining the positioning of the first and last nucleosome inside the coding region. In addition, the formation of evenly spaced nucleosomal arrays may also be affected by the high AT-content of the *P. falciparum* genome. Instead of being placed at a fixed distance from the neighboring nucleosome, nucleosomes may preferentially locate to regions with the least inhibitory sequences, resulting in seemingly more random spacing. Despite these differences in nucleosome organization around coding regions, our results suggest that the general relation between nucleosome occupancy and promoter strength that has previously been uncovered in yeast also applies to *P. falciparum*.

### Global nucleosome dynamics associated with transcriptional activity

The dramatic genome-wide changes in chromatin organization during the trophozoite stage are in stark contrast with the modest rearrangements in nucleosome positioning that are generally observed during transcription in eukaryotes. Our observations in *P. falciparum* are consistent with a model in which genome-wide nucleosome eviction at the trophozoite stage drives massive transcriptional activity. This global change in chromatin structure is confirmed by a recent study that determined the three-dimensional structure of the parasite genome throughout its erythrocytic cycle, demonstrating the opening of chromatin at the trophozoite stage [43]. In the absence of a large variety of transcription factors, nucleosome occupancy levels of individual promoters and gene regions may fine-tune the level of transcription for each gene by carefully regulating the accessibility of promoter regions to more general transcription factors and the RNA polymerase II machinery. Interestingly, we observed that sexual genes have more densely nucleosome-packed promoters that may prevent activation of these genes during the asexual cell cycle. Nucleosome repackaging during the schizont stage reduces the transcriptionally permissive state of the genome, and prepares the parasite for the process of egress and re-invasion. During stages with higher nucleosome occupancy, stage-specific gene expression may be tightly controlled by local chromatin remodeling complexes that provide access of highly selective transcription factors [44] to promoter regions. Thus, nucleosome remodeling and specific transcription factors are likely to act in concert to regulate both the levels and the timing of gene expression.

Nucleosome depletion during the trophozoite stage could also be related to DNA replication, which also occurs during this stage. However, DNA replication is generally associated with an increased level of histones in eukaryotic cells (reviewed in [45]). The mechanisms employed by *P. falciparum* to organize its genome-wide nucleosome eviction remain to be fully described. The *P. falciparum* genome is known to encode a relatively large amount of chromatin remodeling enzymes [16], and many more may be present among its approximately 1,600 conserved genes for which the function is currently unknown. Considering the potential importance of nucleosome remodeling for cell cycle progression, parasite-specific enzymes that are involved in this process may be prime targets for novel antimalarial drugs.

### Histone modifications and variants

Nucleosome occupancy is likely to act in concert with other mechanisms to form a regulatory network that controls gene expression at multiple levels. In other eukaryotes, both histone variants and histone post-translational modifications influence chromatin structure and are important contributors to gene regulation. In the malaria parasite, this correlation is less conserved. *P. falciparum* histones are predominantly acetylated and monomethylated resulting in a transcriptionally permissive state [46], while heterochromatin marked by H3K9me3 and H3K36me3 is restricted to regions that contain tightly regulated gene families, such as *var*, *rif*, and *stevor*[47, 48]. Similarly, H2A.Z is mainly found in the +1 nucleosome of active promoters in other eukaryotes, while the universal localization of H2A.Z to intergenic regions in *P. falciparum*[40, 41] points towards a differential function of this histone variant. The correlation between nucleosome occupancy and transcript levels as described in this study is at least as strong as what has been observed for any histone modification or variant. We therefore speculate that nucleosome occupancy itself could be a major determinant of gene expression levels in *P. falciparum*. However, it will be necessary to define the role of all epigenetic mechanisms of gene regulation to completely understand transcriptional regulation in this parasite.

## Conclusions

The results of this study suggest that nucleosome positioning in *P. falciparum* is strongly influenced by DNA sequence composition and that chromatin organization is an important regulator of gene expression levels. A deeper understanding of chromatin structure and nucleosome dynamics involved in parasite-specific mechanisms of gene regulation could contribute to the discovery novel anti-malarial drug targets.

## Methods

### Parasite culture

*P. falciparum* strains 3D7 and NF54 were cultured in white blood cell-depleted human O^+^ erythrocytes at 5% hematocrit as previously described [49]. For asexual time points, 3D7 cultures were synchronized twice at ring stage with 5% D-sorbitol treatments performed eight hours apart [50]. Parasite developmental stages were assessed by Giemsa-stained blood smears. Cultures at 8% parasitemia were harvested 48 hours after the first sorbitol treatment (ring stage), and then 18 hours (trophozoite stage) and 36 hours thereafter (schizont stage). For the MNase ChIP-Seq experiment, ring stage cultures were obtained early in the next erythrocytic cycle, after re-invasion of parasites (6 hours after the schizont stage). The induction of gametocyte-stage parasites in the NF54 cell line was adapted from a previously published protocol [51]. In brief, parasites were synchronized by 5% sorbitol lysis and diluted the following day into 75 cm^2^ flasks to reach 0.5% parasitemia at a hematocrit of 8.3% (total volume of 15 ml). Parasites were stressed for 3 days by daily replacement of 10 ml of culture media. Cultures with a 5-10% parasitemia were then induced by increasing media to a final volume of 25 ml per flask. For the next 5 days, cultures were maintained by removing 10 ml of media and adding 10 ml of fresh media supplemented with 50 mM N-acetyl glucosamine (NAG) to extinguish asexual parasites. Subsequently, cultures were fed with regular media until harvest of gametocytes at 2% parasitemia 14 days after induction, corresponding to stage IV-V of gametocytogenesis. MAINE-Seq [18] and RNA-Seq [29] were performed as described previously. ChIP-Seq was performed as described below.

### Parasite extractions

Parasites cultures were pelleted for 5 min at 660 × g at 4°C and subsequently lysed by 10 min incubation on ice in 0.15% saponin in water. Parasites were then centrifuged for 10 min at 4,211 × g at 4°C, resuspended in PBS, centrifuged for 10 min at 2,000 × g at 4°C, resuspended in PBS, transferred to a microcentrifuge tube and centrifuged for 5 min at 5,000 rpm at RT in a microcentrifuge. Subsequently, parasites were crosslinked for 5 min (MNase protocol) or 10 min (sonication protocol) in 1% fresh formaldehyde in PBS at RT. The crosslinking reaction was quenched by adding glycine to a final concentration of 0.125 M and incubating at RT for half the incubation time used for crosslinking. Parasites were centrifuged at for 5 min at 5,000 rpm at 4°C and washed twice in cold PBS before parasite pellets were stored at -80°C. Parasite were resuspended in nuclear extraction buffer (10 mM HEPES, 10 mM KCl, 0.1 mM EDTA, 0.1 mM EGTA, 1 mM DTT, 0.5 mM 4-(2-aminoethyl)benzenesulfonyl fluoride hydrochloride (AEBSF), and EDTA-free protease inhibitor cocktail (Roche, Basel, Switzerland)) and incubated for 30 min on ice. Igepal CA-360 (Sigma-Aldrich, St. Louis, MO) was added to a final concentration of 0.25%, followed by mechanical lysis of parasites by passing the suspension ten times through a 26 G ½ inch needle. Parasite nuclei were then centrifuged for 20 min at 5,000 rpm at 4°C.

### Chromatin fragmentation by sonication

Parasite nuclei were resuspended in SDS lysis buffer (1% SDS, 10 mM EDTA, 50 mM Tris-HCl pH 8.1, and EDTA-free protease inhibitor cocktail), diluted to a concentration equivalent to 0.1 μg/μl DNA, and distributed over 1.5 ml TPX polymethylpentene tubes (300 μl per tube; Diagenode, Denville, NJ). Suspensions were sonicated for a total of 25 cycles of 30 s ON and 30 s OFF at high intensity using a Bioruptor UCD-200 (Diagenode). Samples were centrifuged for 10 min at 14,000 rpm at 4°C to remove insoluble material, and fragmented chromatin was stored at -80°C until further use.

### Chromatin fragmentation by MNase treatment

Parasite nuclei were resuspended in MNase buffer (50 mM Tris-HCl pH 7.4, 4 mM MgCl_2_, 1 mM CaCl_2_, 2 mM AEBSF, and EDTA-free protease inhibitor cocktail) and transferred to 1.5 ml TPX polymethylpentene tubes. Nuclei were permeabilized by mild sonication for 4 cycles of 15 s ON and 45 s OFF at high intensity using a Bioruptor UCD-200. Next, 50 U MNase (USB corporation, Cleveland, OH) was added, followed by incubation for 5 min at 37°C. MNase was inactivated by the addition of EDTA to a final concentration of 5 mM and incubation for 5 min at RT with agitation. The samples were diluted 10x in immunoprecipitation buffer (15 mM Tris-HCl pH 8.1, 150 mM NaCl, 1.5 mM EDTA, 1% Triton X-100, 0.1% SDS, and EDTA-free protease inhibitor cocktail), incubated for 10 min at 4°C, and centrifuged for 5 min at 14,000 rpm at 4°C to remove insoluble material. Fragmented chromatin was stored at -80°C until further use.

### Chromatin immunoprecipitation

Chromatin fragmented by sonication was first diluted 10x in ChIP dilution buffer (0.01% SDS, 1% Triton X-100, 1.2 mM EDTA, 16.7 mM Tris-HCl pH 8.1, 167 mM NaCl, and EDTA-free protease inhibitor cocktail). Immunoprecipitations were then performed using the Chromatin Immunoprecipitation Assay Kit (Millipore, Billerica, MA) according to the manufacturer’s protocol. In brief, samples were precleared with Protein A Agarose beads to reduce non-specific background and were then incubated O/N at 4°C with anti-H3 antibody raised against a peptide within the region between residue 100 and the C-terminus that is identical between H3 and H3.3 (ab1791, Abcam, Cambridge, UK), an anti-H4 antibody (05-858, Millipore) or a non-specific antibody (custom-made antibody directed against a putative ubiquitin-activating enzyme E1 [PF3D7_1225800]). Immunocomplexes were recovered using Protein A Agarose beads, followed by extensive washes in low salt immune complex wash buffer, high salt immune complex wash buffer, LiCl immune complex wash buffer and finally TE buffer. Chromatin was eluted from the antibody by two subsequent incubations of 15 min at RT in freshly prepared elution buffer (1% SDS, 0.1 M NaHCO_3_). To reverse crosslinking, NaCl was added to a final concentration of 0.5 M, followed by O/N incubation at 45°C. Samples were then treated with RNase A (Life Technologies, Carlsbad, CA) for 30 min at 37°C. EDTA (final concentration 8 mM), Tris-HCl pH6.5 (final concentration 33 mM) and proteinase K (final concentration 66 μg/ml; New England Biolabs, Ipswich, MA) were added, followed by incubation for 2 h at 45°C. DNA was recovered by phenol:chloroform:isoamylalcohol extraction and ethanol precipitation. DNA was further purified using Agencourt AMPure XP beads (Beckman Coulter, Brea, CA).

### Library preparations and sequencing

Libraries from sonication ChIP samples were prepared using the Encore Multiplexing System (NuGEN, San Carlos, CA) according to the manufacturer’s instructions, with the following modifications for the high AT-content of the *P. falciparum* genome: libraries were amplified for a total of 15 PCR cycles (5 cycles of [15s at 98°C, 30s at 55°C, 30s at 62°C] followed by 10 cycles of [15 s at 98°C, 30s at 63°C, 30s at 72°C]) using KAPA HiFi HotStart Ready Mix (Kapa Biosystems, Woburn, MA). Libraries from MNase ChIP samples were prepared using the NEBNext ChIP-Seq Library Preparation kit (NEB) according to the manufacturer’s instructions, with the following modifications for the high AT-content of the *P. falciparum* genome: libraries were amplified for a total of 11 PCR cycles (3 cycles of [15 s at 98°C, 30s at 55°C, 30s at 62°C] followed by 8 cycles of [15 s at 98°C, 30s at 63°C, 30s at 72°C]) using KAPA HiFi HotStart Ready Mix. Libraries were sequenced with a HiSeq 2000 (Illumina, San Diego, CA), generating 50 bp paired-end sequence reads. Sequence reads are available through the Short Read Archive under BioProject numbers SRP026365 (nucleosome positioning data) and SRP026367 (steady-state mRNA data gametocytes). Steady-state mRNA sequence data from ring, trophozoite and schizont stages are available under accession numbers SRS417027, SRS417268, and SRS417269, respectively.

### Sequence mapping

The first 5 bases and the last base were systematically removed from the sequence reads using FastQ Trimmer, part of the FASTX-Toolkit (http://hannonlab.cshl.edu/fastx_toolkit/index.html). Contaminating adaptor reads were removed using Scythe (https://github.com/ucdavis-bioinformatics/scythe). Reads were then trimmed for bases with a quality score below 30, and reads containing any Ns as well as reads shorter than 18 bases were discarded using Sickle (https://github.com/ucdavis-bioinformatics/sickle). The trimmed sequence reads were first mapped to the human genome (HG19, downloaded from ftp://ftp.1000genomes.ebi.ac.uk/vol1/ftp/), and all non-mapped reads were subsequently mapped to *P. falciparum* 3D7 genome v9.0 (downloaded from http://www.plasmoDB.org) using BWA [52] with default error rates, allowing a maximum of 1500 bp distance between read pairs. Any read that was either non-uniquely mapped (Samtools v0.1.18 [53]), not properly paired (Samtools) or a PCR duplicate (Picard Tools v1.78 [http://picard.sourceforge.net/]) was discarded. The final number of mapped reads for each library is listed in Additional file 1: Table S1.

### Nucleosome positioning and normalization strategy

Nucleosome positions and score were computed using our novel nucleosome positioning software tool PuFFIN [27]. PuFFIN is a multi-scale peak-calling method that replaces each nucleosome-enriched paired-end sequence read with a Gaussian curve. By summing these Gaussian distributions for all paired-end reads, PuFFIN creates a smoothed coverage function, in which each peak represents a candidate nucleosome location. By changing the width of the Gaussian curves, PuFFIN can capture candidate locations at different resolution scales. The number of reads mapped to a nucleosome (expressed per 10^7^ of total mapped reads) was used as a score for nucleosome strength.

To compare nucleosome levels between time points, nucleosome scores were normalized by first calculating the ratio between the number of reads mapped to the *P. falciparum* genome and the total number of reads mapped to both the human and the *P. falciparum* genome. The final normalization factor was obtained by dividing the normalization ratio of each stage by the normalization ratio of the ring stage. Normalization ratios and final normalization factors are shown in Additional file 1: Table S2. The rationale behind this normalization strategy is that the amount of human DNA contamination is assumed to be relatively constant (see below), while the amount of nucleosome-bound *P. falciparum* DNA recovered after immunoprecipitation is highly influenced by nucleosome levels in the parasite. We therefore expect the fraction of sequence reads mapped to the *P. falciparum* genome out of the total number of mapped reads to be directly proportional to nucleosome occupancy. Various factors complicate the comparison of nucleosome levels at different stages: (1) the ratio of human versus *P. falciparum* DNA changes during the cell cycle as the parasite multiplies its genome while the amount of human cells is invariant. Accordingly, the fraction of human sequence reads at the schizont stage of the asexual cell cycle is lower as compared to the ring stage. (2) Gametocyte cultures are harvested at relatively low parasitemia after two weeks of maturation and are thus expected to have smaller numbers of viable human white blood cells. (3) The level of human DNA contamination may differ from experiment to experiment, depending on the amount human white blood cells in culture and subsequent experimental methodologies. As a result of its non-linear relationship and inter-experimental variability, the true ratio between human and *P. falciparum* sequence reads is very difficult (if not impossible) to model. We therefore assumed this ratio to be constant in each sample. Our normalization strategy may result in a slight overestimation of nucleosome levels at the schizont stage, but the lower-than-expected percentage of *P. falciparum* sequence reads at the trophozoite stage (Additional file 1: Table S2) in our study, as well as in previously published nucleosome occupancy sequencing data [20], clearly demonstrates reduced nucleosome levels at this time point. Normalization using only *P. falciparum*-mapped sequence reads does not allow a quantitative assessment of nucleosome levels, but rather eliminates all information about its effective library size. Of note, our normalization strategy only impacts the comparison of nucleosome levels between time points. The normalization factor is applied in a genome-wide fashion, and therefore does not influence the results of analyses within each sample (*i.e.* differences in nucleosome landscape between subsets of genes).

### Nucleosome positioning analysis in transcription clusters

Since transcription start sites have not been annotated genome-wide and the majority of annotated transcription start sites in the compact *P. falciparum* genome are located within 500 bp of the translation start site, we used the 500 base pair region directly upstream of the translation start codon to compute nucleosome characteristics of promoter regions. To avoid overlap of these promoter regions with gene regions, only genes that are located more than 500 nucleotides away from the neighboring gene were included in the analysis (excluding 136 genes). In addition, gene families which may be subject to alternative mechanisms of transcriptional regulation were excluded (*var*, *rifin*, *stevor*, *surfin*, *Pfmc-2tm*; *n* = 331). Of the remaining 5036 genes, the 36 genes with lowest expression values were also discarded to obtain a group of 5000 genes that could easily be divided into equally sized groups.

Transcription start sites have been experimentally determined approximately 50% of *P. falciparum* genes [54, 55]. Non-overlapping genes with a known TSS [19] that were located at least 500 bp from the neighboring gene were used to plot the sequence coverage around the TSS (see “Coverage plots”).

The average number of nucleosomes and the average nucleosome scores per transcription cluster were calculated by first computing the average value for each gene, and then computing the average value for all genes in each gene cluster.

Genes with stage-specific expression profiles were selected based on transcription clusters reported in a previous study [29]. Genes that were identified as being expressed at a single stage of the asexual cell cycle (either ring, trophozoite, or schizont stage) and that were expressed at relatively low levels at the gametocyte stage (bottom 70% expression levels of all genes) were included in the stage-specific expression groups. Gametocyte-stage specific expressed genes were defined as all genes among the top 30% expression levels at the gametocyte stage and the bottom 50% expression levels in all other stages.

### Analysis of previously published data sets

*P. falciparum* 3D7 nucleosome positioning single-end sequence reads [20] were downloaded (SRX026772/3) and trimmed using the same strategy as used for sequence read libraries generated in this study. Sequence reads were then mapped to *P. falciparum* 3D7 genome version 9.0 (http://www.plasmodb.org) using BWA with default error rates. Based on Giemsa-stained blood smears provided with the publication, the sample obtained 30 hours post-invasion was determined to represent the trophozoite stage. For the generation of nucleosome maps, single-end reads were extended to 110 bp and nucleosome positions were subsequently determined using the PuFFIN software. The score for each nucleosome was normalized as described in the section “Nucleosome positioning and normalization strategy”.

*Saccharomyces cerevisiae* nucleosome positioning sequence reads [31] were downloaded (SRA001003) and were mapped to the *S. cerevisiae* BY4741 genome version Toronto_2012 (downloaded from http://www.yeastgenome.org/) using bowtie2 with “—very-fast-local” setup to account for possible adapter contamination. An MNase-Seq nucleosome positioning data set for the human cell line GM12878 was downloaded from the ENCODE database (DCC Accession Number wgEncodeEH000922) [33]. Nucleosome positions and scores were determined using PuFFIN. Analysis of nucleosome positioning in relation to transcription levels were performed as described above for *P. falciparum*, using a previously published RNA-Seq expression data set for *S. cerevisiae*[32] and an RNA-Seq expression data set for the human cell line GM12878 generated as part of the ENCODE project (file wgEncodeCaltechRnaSeqRawData5Rep1Gm12878CellLongpolyaErng32x75.rpkm) for human [33].

### Coverage plots

Genome browser plots were obtained using Artemis [56]. Coverage plots were prepared by extracting the read counts for the region of interest for all genes included in the analysis, and subsequently calculating the average value for each nucleotide position. Coverage profiles were smoothed using overlapping 150 bp sliding windows, and were subsequently plotted using bioconductor R package ggplot2. For coverage heatmaps, read counts per nucleotide were divided by the total number of mapped reads (× 10^7^) and subsequently multiplied by the stage-specific normalization factors (Additional file 1: Table S2). Nucleotide positions with zero read coverage were replaced with the lowest overall read coverage (i.e. 0.001). Read counts were then converted to log 10 values, and were finally plotted in R using the pheatmap package.

### Analysis of nucleosome binding preferences

For both intergenic and coding regions, enrichment of 5-mers was calculated as the log base 2 ratio of the frequency of 5-mers within 147 bp nucleosome-bound DNA fragments (based on intergenic and genic nucleosome positions reported by PuFFIN +/- 73 bp) and the 5-mer frequency in all intergenic regions and coding regions, respectively. To identify differences in sequence composition between genes with different expression levels, all 1024 possible 5-mers were divided into five groups based on their genome-wide frequency in intergenic or coding regions. Subsequently, the average 5-mer enrichment in promoter and coding regions was calculated for each transcription cluster of 500 genes and was z-normalized across the five frequency groups.

### Availability of supporting data

The data sets supporting the results of this article are available in the Short Read Archive, [SRP026365, SRP026367, SRS417027, SRS417268, SRS417269; http://www.ncbi.nlm.nih.gov/sra].

## Electronic supplementary material

Additional file 1: Figure S1: Schematic overview of nucleosome positioning methodologies. **Figure S2.** Distributions of fragment sizes of Sonication ChIP‒Seq and MNase ChIP‒Seq libraries. **Figure S3.** High correlation between different nucleosome mapping data sets. **Figure S4.** Adjusted number of nucleosomes after correcting for differences in sequencing library size. **Figure S5.** Percentage of sequence reads mapped to genes or intergenic regions. **Figure S6.** Nucleosome mapping results for sonication ChIP‒Seq samples. **Figure S7.** Association between nucleosome occupancy and transcription level at the trophozoite stage. **Figure S8.** Open chromatin structure at the transcription start sites of highly expressed genes. **Figure S9.** Correlations between nucleosome occupancy and transcription level for other publicly available RNA‒Seq data sets. **Figure S10.** Correlations between nucleosome occupancy and transcription level for transcription clusters of 50 genes each. **Figure S11.** Correlations between nucleosome occupancy and transcription level for randomly generated clusters of 50 genes each. **Figure S12.** Correlations between nucleosome occupancy and transcription level in *S. cerevisiae* and human cells. **Figure S13.** Associations between nucleosome occupancy and transcription level. **Figure S14.** Binding preferences of nucleosomes located in genes and intergenic regions. **Figure S15.** Distribution of nucleosome‒disfavoring sequences in *P. falciparum* nucleosomes. **Figure S16.** Association between sequence composition and transcription levels. **Table S1.** Overview of sequence reads mapped to the human and *P. falciparum* genomes. **Table S2.** Normalization factors. (PDF 1 MB)

## Abbreviations

MNase: Micrococcal nuclease
NDR: Nucleosome-depleted region
TSS: Transcription start site
Bp: Base pair
MAINE: MNase-assisted isolation of nucleosome-bound DNA
FAIRE: Formaldehyde-assisted isolation of protein-free DNA
ChIP: Chromatin immunoprecipitation.
