# Supplementary material for: DNA-encoded nucleosome occupancy is associated with transcription levels in the human malaria parasite Plasmodium falciparum
Source: BMC Genomics. 2014 May 8;15(1):347. doi: 10.1186/1471-2164-15-347 (PMC4035074; doi:10.1186/1471-2164-15-347)
Supplement: Supplementary file 1 — Additional file 1: Figure S1: Schematic overview of nucleosome positioning methodologies. Figure S2. Distributions of fragment sizes of Sonication ChIP‒Seq and MNase ChIP‒Seq libraries. Figure S3. High correlation between different nucleosome mapping data sets. Figure S4. Adjusted number of nucleosomes after correcting for differences in sequencing library size. Figure S5. Percentage of sequence reads mapped to genes or intergenic regions. Figure S6. Nucleosome mapping results for sonication ChIP‒Seq samples. Figure S7. Association between nucleosome occupancy and transcription level at the trophozoite stage. Figure S8. Open chromatin structure at the transcription start sites of highly expressed genes. Figure S9. Correlations between nucleosome occupancy and transcription level for other publicly available RNA‒Seq data sets. Figure S10. Correlations between nucleosome occupancy and transcription level for transcription clusters of 50 genes each. Figure S11. Correlations between nucleosome occupancy and transcription level for randomly generated clusters of 50 genes each. Figure S12. Correlations between nucleosome occupancy and transcription level in S. cerevisiae and human cells. Figure S13. Associations between nucleosome occupancy and transcription level. Figure S14. Binding preferences of nucleosomes located in genes and intergenic regions. Figure S15. Distribution of nucleosome‒disfavoring sequences in P. falciparum nucleosomes. Figure S16. Association between sequence composition and transcription levels. Table S1. Overview of sequence reads mapped to the human and P. falciparum genomes. Table S2. Normalization factors. (PDF 1 MB) [file 12864_2014_6037_MOESM1_ESM.pdf]

Supplementary Figures and Tables to:

DNA-encoded nucleosome occupancy regulates transcription levels in the human malaria parasite *Plasmodium falciparum*

Evelien M. Bunnik[1], Anton Polishko[2], Jacques Prudhomme[1], Nadia Ponts[1,3], Sarjeet S. Gill[1], Stefano Lonardi[2] and Karine G. Le Roch[1]*

[1] Department of Cell Biology and Neuroscience, University of California, Riverside, Riverside, CA 92521, USA

[2] Department of Computer Science and Engineering, University of California, Riverside, Riverside, CA 92521, USA

[3] Present Address: Mycology and Food Safety, INRA Centre de Bordeaux-Aquitaine, Villenave d'Ornon Cedex, 33883, France

* Corresponding author. Email: karine.leroch@ucr.edu

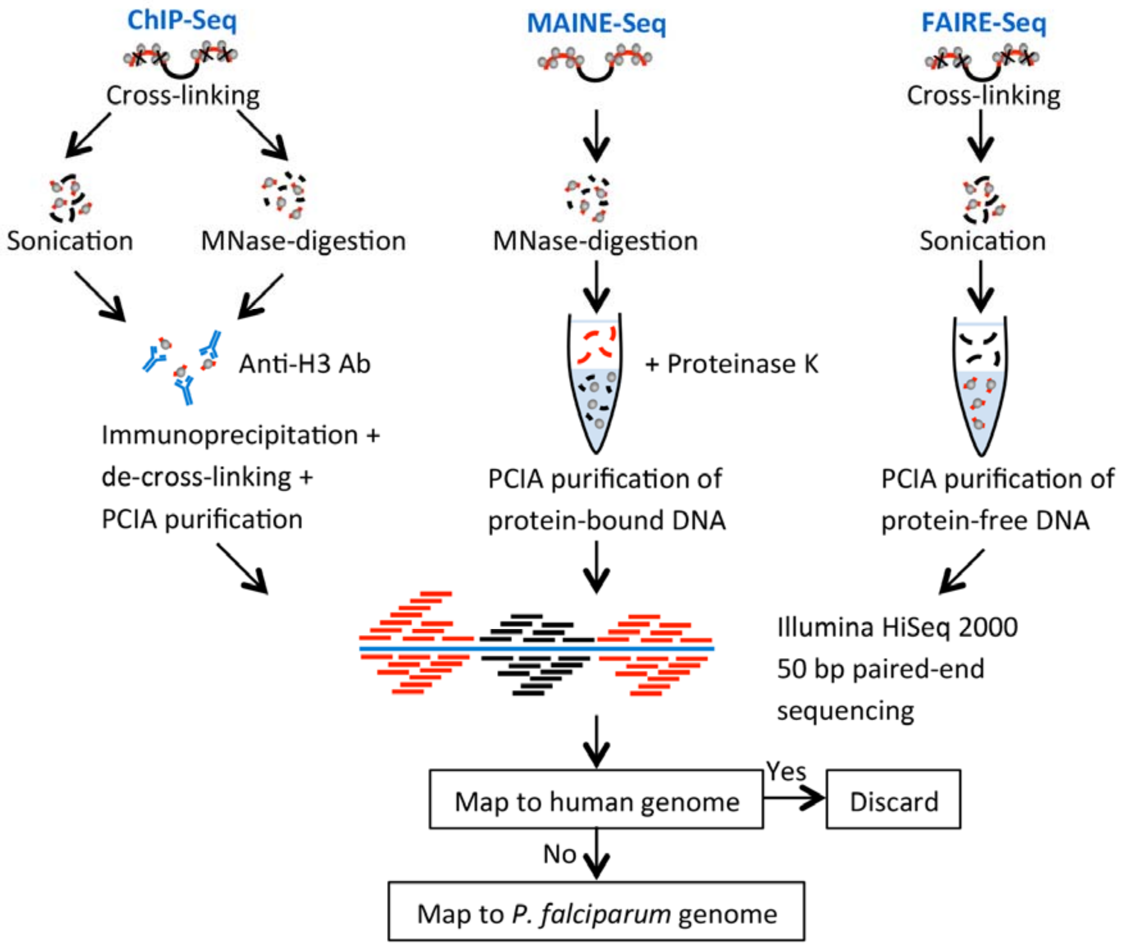

Figure S1. Schematic overview of nucleosome positioning methodologies. In this study, the nucleosome landscape during the asexual cell cycle of the human malaria parasite was analyzed using ChIP-Seq. Chromatin was cross-linked using formaldehyde and fragmented by either sonication or MNase-treatment. Nucleosomes were subsequently immunoprecipitated using an antibody directed against histone H3, followed by DNA purification. Nucleosome-bound DNA fragments were analyzed by next-generation sequencing (Illumina HiSeq 2000), generating 50 bp paired-end sequence reads. Reads were first mapped to the human genome to eliminate contamination, followed by mapping of all remaining reads to the *P. falciparum* genome. Nucleosome positioning at the gametocyte stage was analyzed by MAINE-Seq, which involves digestion of chromatin using MNase and purification of nucleosome-protected DNA fragments. As a reference, an overview of the FAIRE-Seq protocol (assessing nucleosome-free DNA) as performed in a previous study (18) is also provided. Ab: antibody; PCIA: phenol-chloroform isoamylalcohol; bp: base pair.

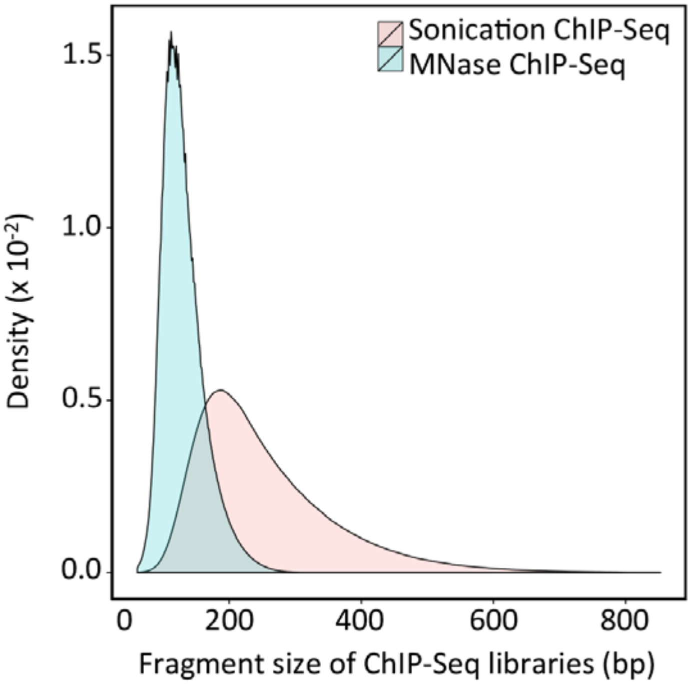

Figure S2. Distributions of fragment sizes of Sonication ChIP-Seq and MNase ChIP-Seq libraries. For MNase-digested chromatin, the average length of sequenced fragments was 130 bp (SD = 32 bp), while sonicated chromatin fragments averaged 253 bp (SD = 104 bp). The shorter than consensus (147 bp) DNA fragment size for MNase-digested chromatin may suggest that nucleosomes in *P. falciparum* are less tightly bound than in other eukaryotic organisms, which could be a result of the high AT-content of its genome.

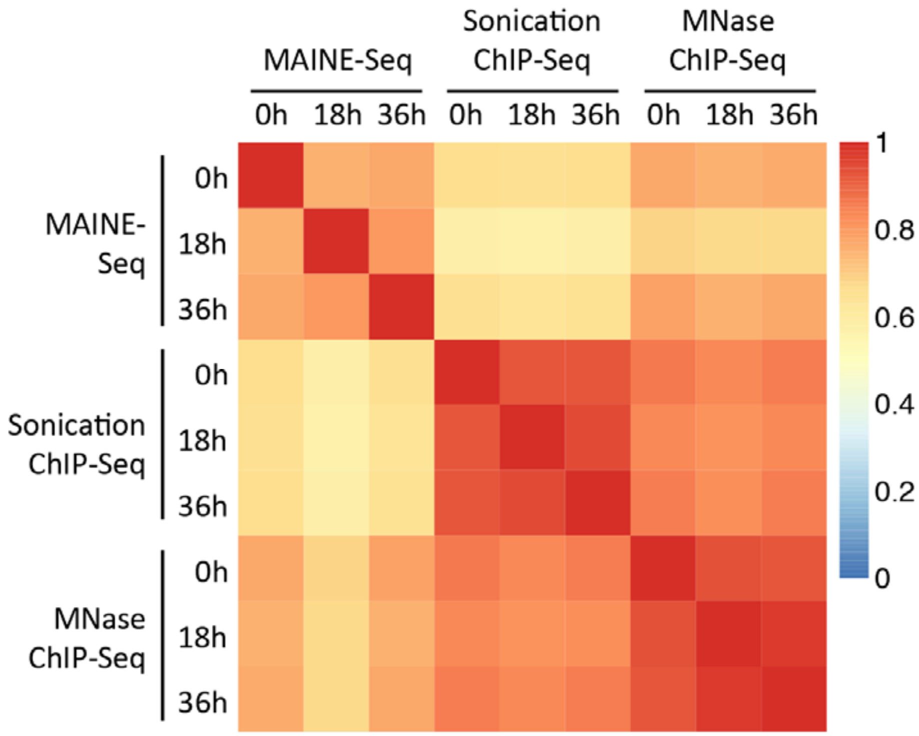

Figure S3. High correlation between different nucleosome mapping data sets. Heatmap of Spearman's rank correlation coefficients between genome coverage of MAINE-Seq (18), Sonication ChIP-Seq and MNase ChIP-Seq samples.

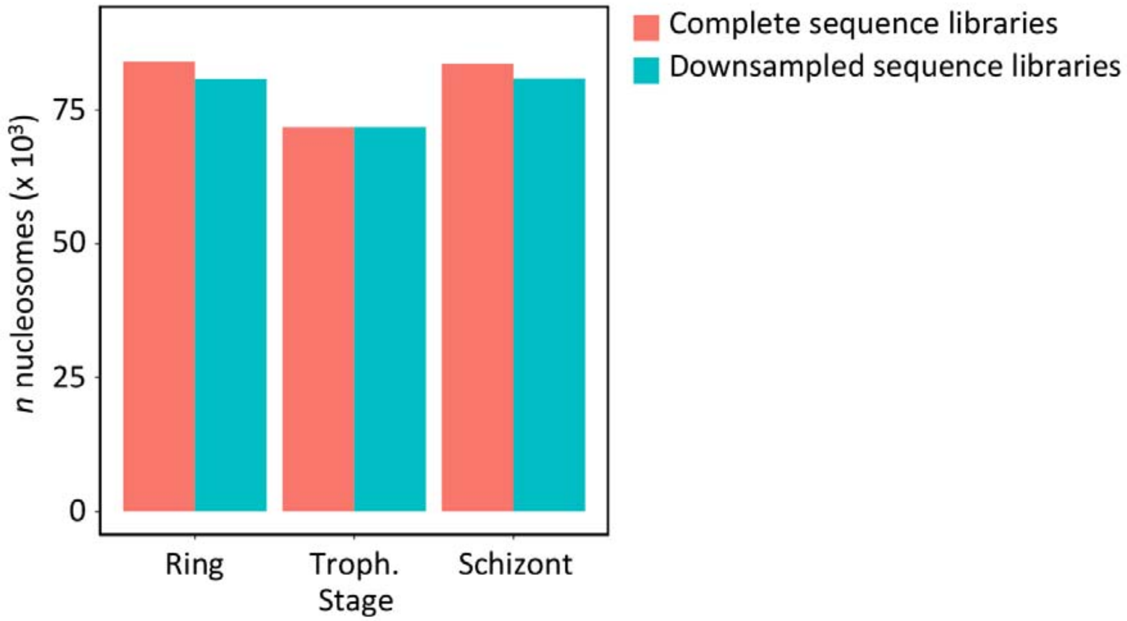

Figure S4. Adjusted number of nucleosomes after correcting for differences in sequencing library size. Since higher sequencing coverage may result in improved detection of nucleosomes, MNase ChIP-Seq libraries were randomly downsampled to the size of the smallest sequencing library (trophozoite stage). The number of nucleosomes detected in the downsampled libraries was slightly reduced, but not to the level of the trophozoite stage, indicating that differences in library size can not account for differences in numbers of nucleosomes detected during the asexual stage. Troph.: trophozoite.

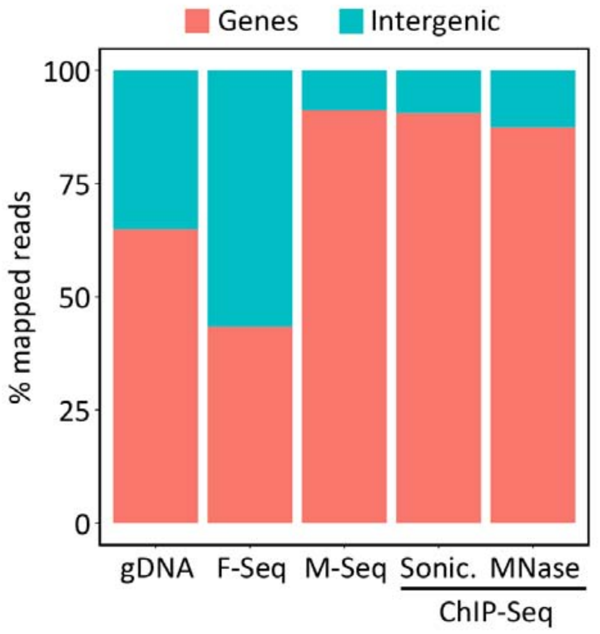

Figure S5. Percentage of sequence reads mapped to genes or intergenic regions. FAIRE-Seq libraries (F-Seq; nucleosome-free DNA (18)) are enriched for sequence reads that map to intergenic regions as compared to genomic DNA (gDNA), indicating that our library preparation and sequencing procedures are capable of sequencing the highly AT-rich intergenic regions of the *P. falciparum* genome. On the other hand, sequencing of nucleosome-bound DNA (MAINE-Seq [M-Seq] (18), Sonication [Sonic.] and MNase ChIP-Seq) resulted in a depletion of sequence reads mapped to intergenic regions and an enrichment of sequence reads mapped to genes, indicative of the differences in nucleosome occupancy between genes and intergenic regions.

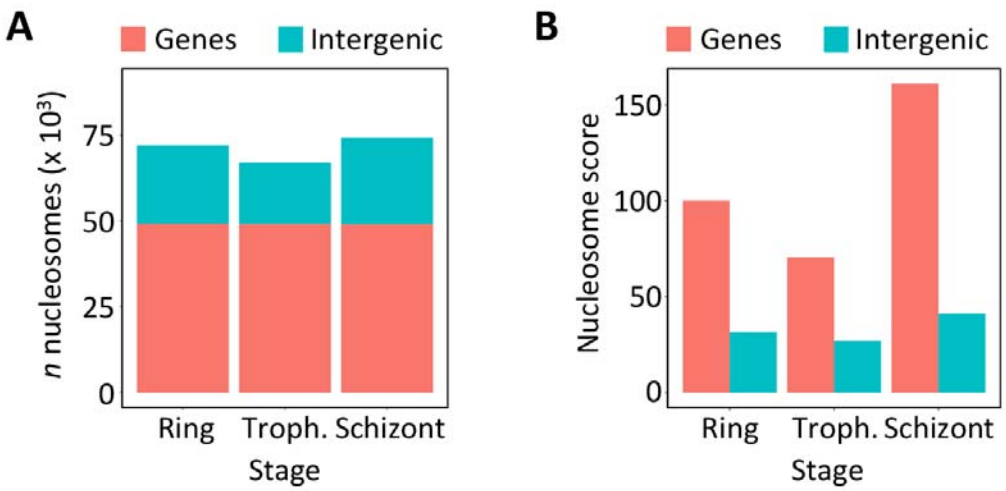

Figure S6. Nucleosome mapping results for sonication ChIP-Seq samples. A. Total number of nucleosomes reported by PuFFIN for different stages of the asexual cell cycle. **B**. Average score of nucleosomes located inside genes and in intergenic regions at different time points of the asexual cell cycle. Values are expressed as the percentage of the average score of nucleosomes located inside genes at the ring stage. Troph.: trophozoite.

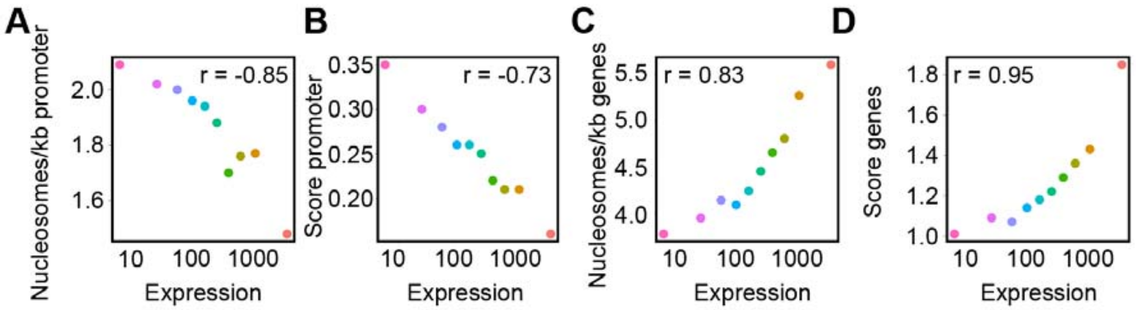

Figure S7. Association between nucleosome occupancy and transcription level at the trophozoite stage. Nucleosome positions and scores were determined using the nucleosome positioning tool NOrMAL (28) with default settings. **A**. The average number of nucleosomes per kilobase in the 500 base pair upstream of the translation start site (promoter region) for each transcription cluster of 500 genes. **B**. The average score of nucleosomes located in the promoter region for each transcription cluster. **C**. The average number of nucleosomes per kilobase inside coding regions for each transcription cluster. **D**. The average score of all nucleosomes located inside coding regions for each transcription cluster. Correlation coefficients reported were obtained using the Spearman's rank test. The relation between the nucleosome landscape and transcript levels is similar for nucleosome maps generated by NOrMAL and PuFFIN, indicating that this result is unlikely to be an artifact of the nucleosome positioning algorithms.

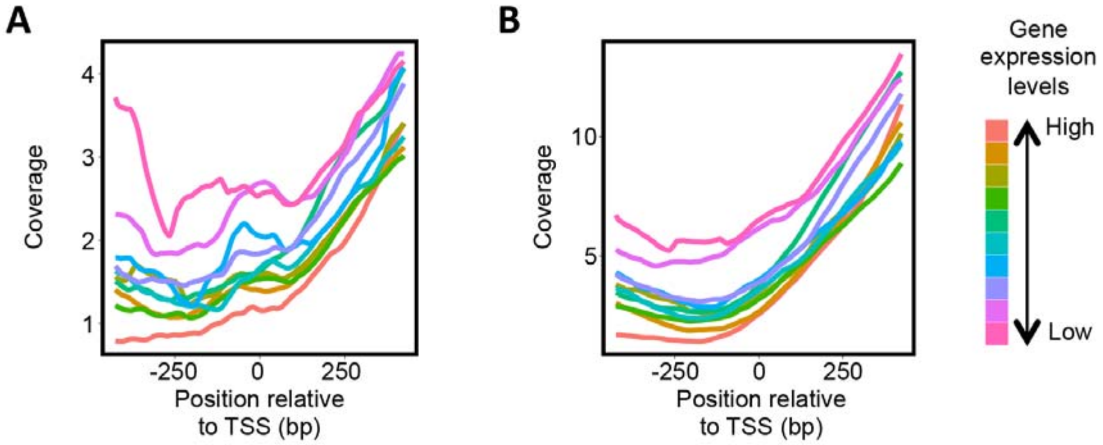

Figure S8. Open chromatin structure at the transcription start sites of highly expressed genes. Plots show the average sequence read coverage around transcription start sites for the subset of genes with annotated transcription start sites in each transcription cluster. Data is shown for sonication ChIP-Seq samples obtained at the trophozoite stage using anti-H3 (**A**) and anti-H4 (**B**) antibodies.

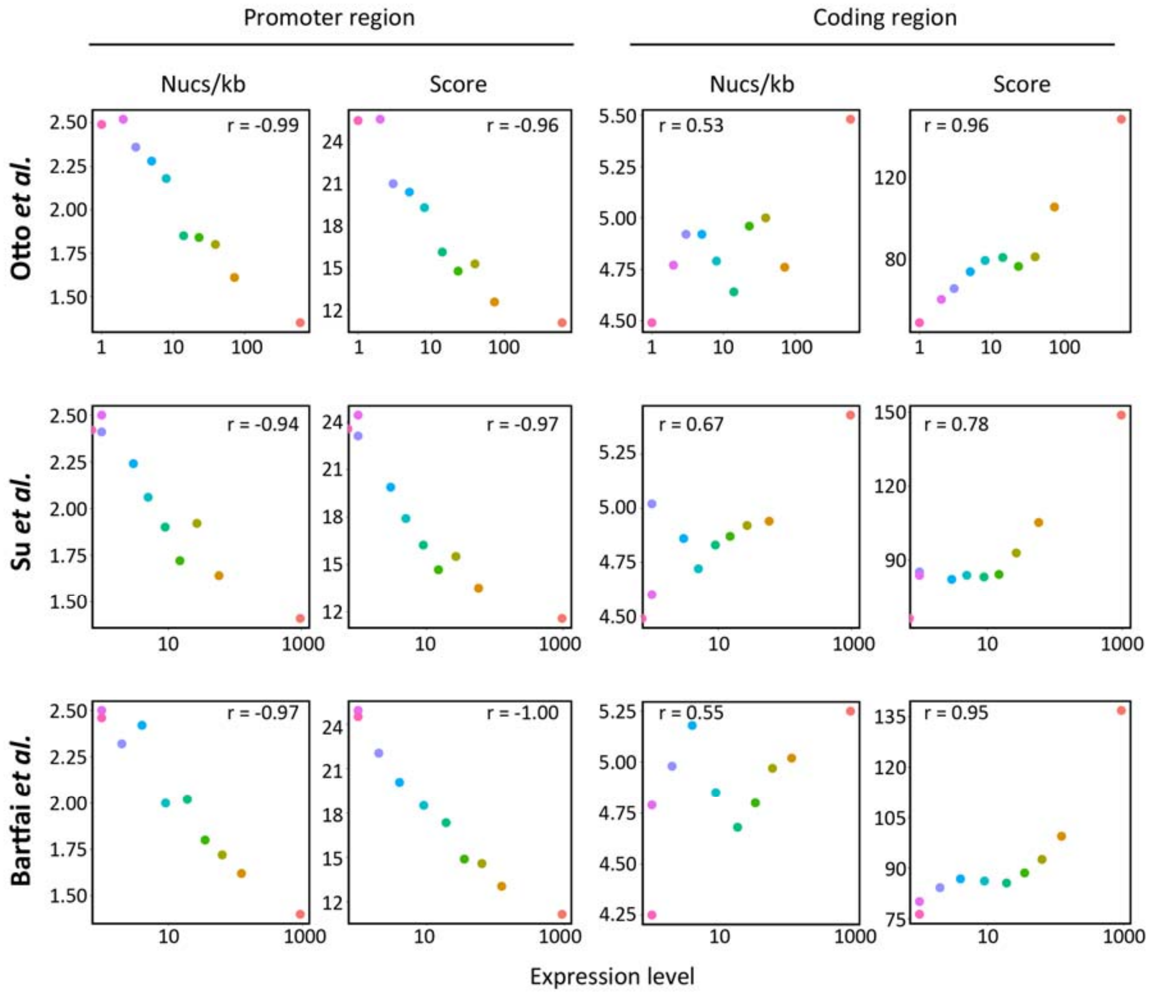

Figure S9. Correlations between nucleosome occupancy and transcription level for other publicly available RNA-Seq data sets. Nucleosome occupancy in the 500 nucleotides upstream of the translation start site (promoter region) and in coding regions is shown for transcription gene clusters at the trophozoite stage. Transcription clusters were computed using RNA-Seq expression levels from Otto *et al.* (26) (top row), López-Barragán *et al.* (30) (middle row), and Bártfai *et al.* (20) (bottom row). RNA-Seq data sets were downloaded from PlasmoDB (www.plasmodb.org). Correlation coefficients reported in the top of each panel were obtained using the Spearman's rank test.

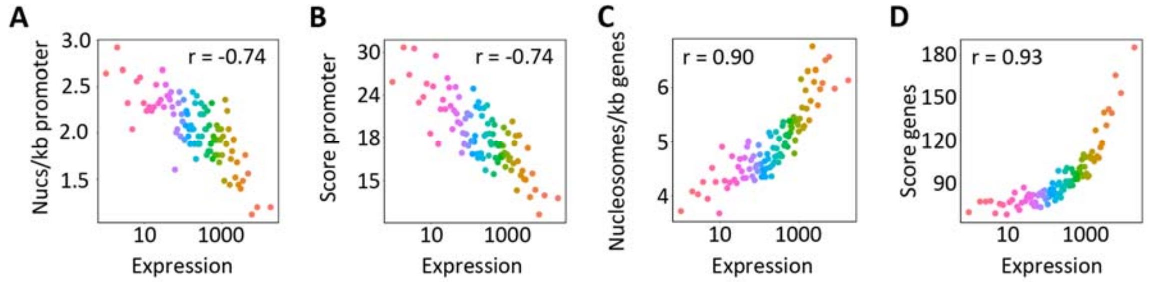

Figure S10. Correlations between nucleosome occupancy and transcription level for transcription clusters of 50 genes each. Nucleosome occupancy in the 500 nucleotides upstream of the translation start site and in coding regions is shown for gene clusters at the trophozoite stage. Correlation coefficients reported in the top of each panel were obtained using the Spearman's rank test.

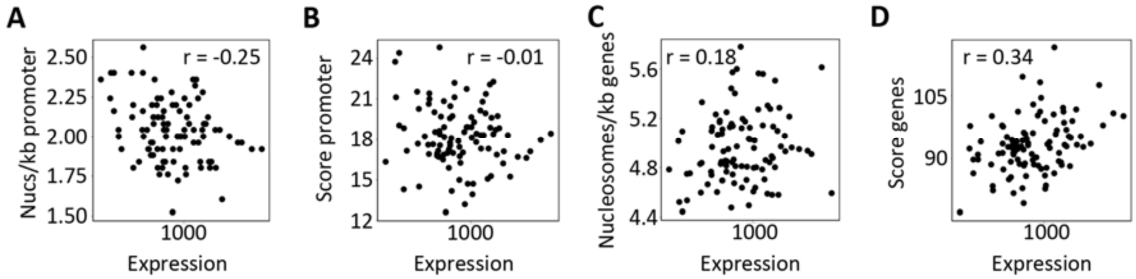

Figure S11. Correlations between nucleosome occupancy and transcription level for randomly generated clusters of 50 genes each. Nucleosome occupancy in the 500 nucleotides upstream of the translation start site and in coding regions is shown for gene clusters using trophozoite stage expression and nucleosome data. Correlation coefficients reported in the top of each panel were obtained using the Spearman's rank test.

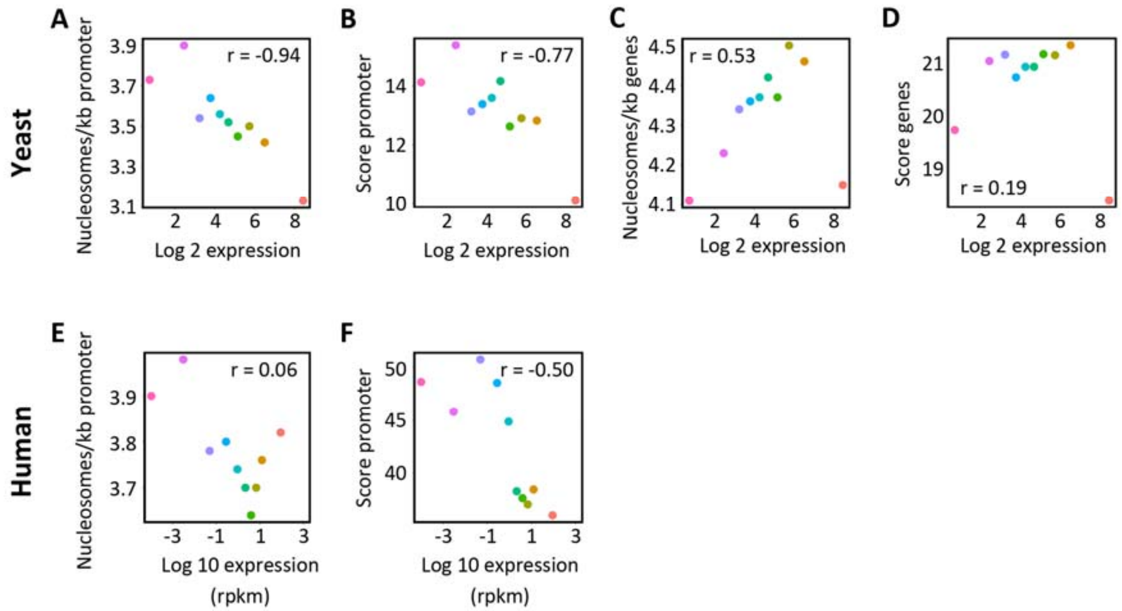

Figure S12. Correlations between nucleosome occupancy and transcription level in *S. cerevisiae* and human cells. A-D. Nucleosome occupancy in the 200 nucleotides upstream of the translation start site (promoter region, panels A and B) and in coding regions (panels C and D) is shown for gene clusters in *S. cerevisiae* using previously published expression (32) and nucleosome (31) data. **E-F.** Nucleosome occupancy in the 500 nucleotides upstream of the transcription start site for gene clusters in human GM12878 cells using nucleosome and transcription data sets generated as part of the ENCODE project (33). Correlation coefficients reported in each panel were obtained using the Spearman's rank test.

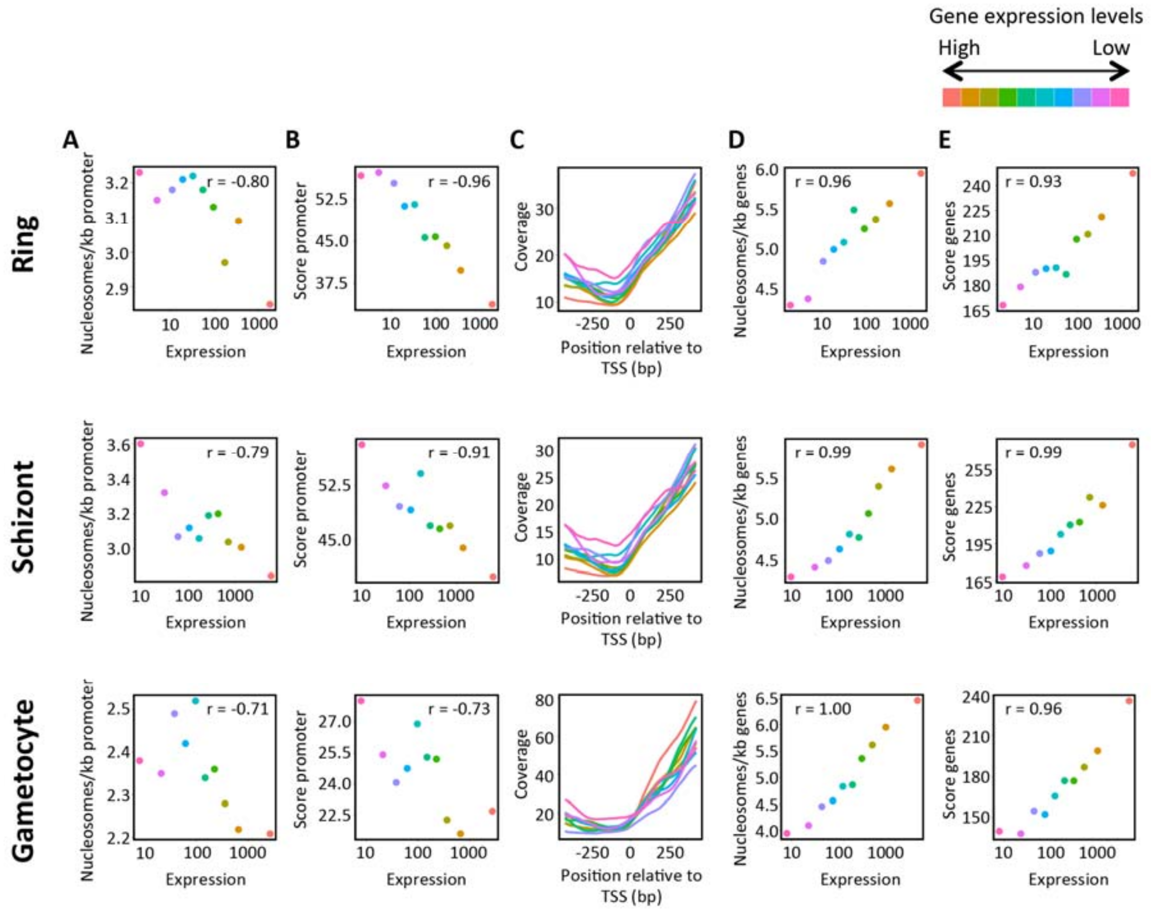

Figure S13. Associations between nucleosome occupancy and transcription level. A. The average number of nucleosomes per kilobase in the 500 base pair upstream of the translation start site (promoter region) for each transcription cluster of 500 genes at the ring (top), schizont (middle) and gametocyte (bottom) stages. **B**. The average score of nucleosomes located in the promoter region for each transcription cluster. **C.** Sequence read coverage around the transcription start site for the subset of genes with annotated transcription start sites in each transcription cluster. **D.** The average number of nucleosomes per kilobase inside coding regions for each transcription cluster. **E.** The average score of all nucleosomes located inside coding regions for each transcription cluster. Correlation coefficients reported were obtained using the Spearman's rank test.

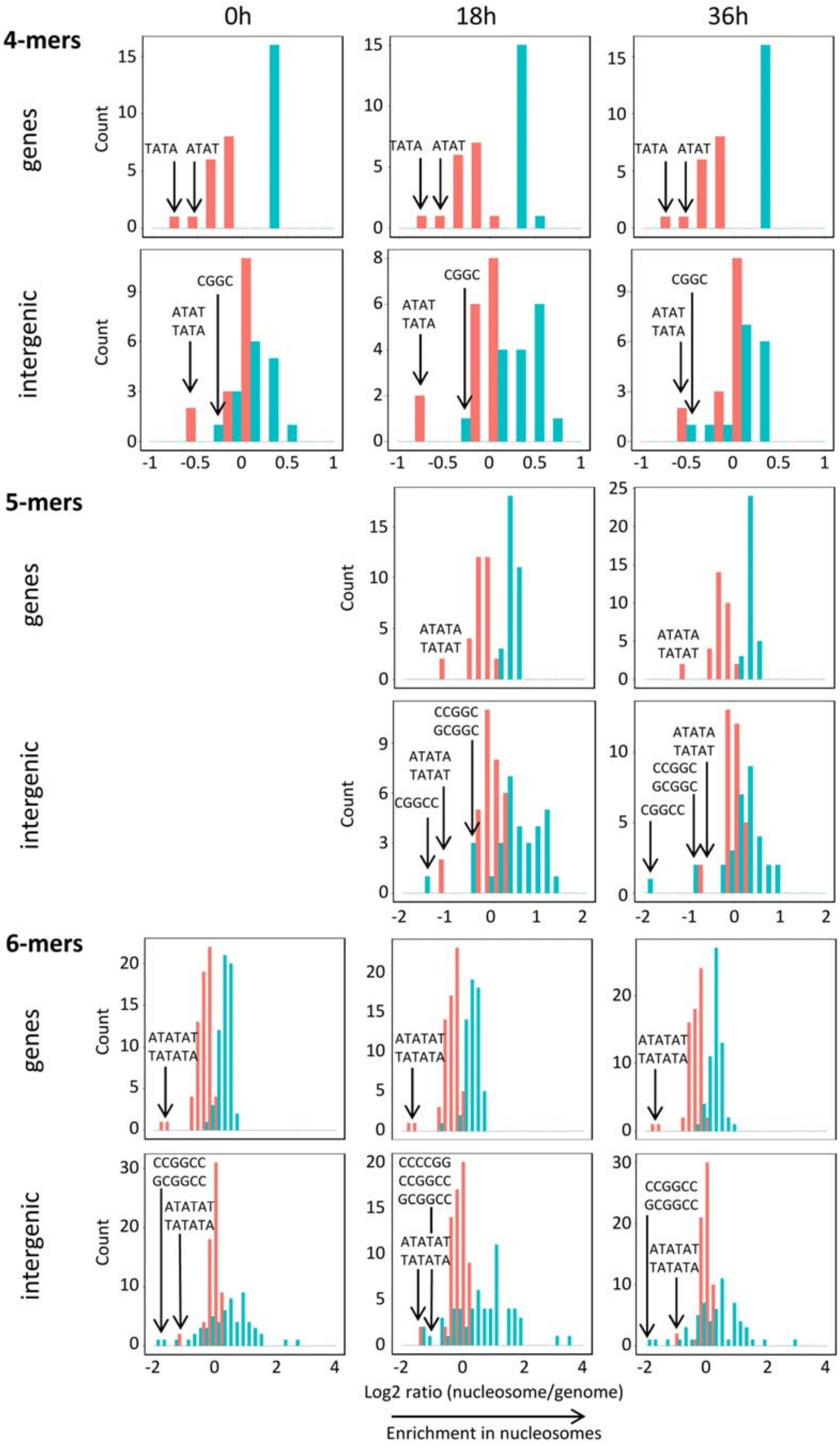

0h
18h
36h
4-mers
genes
intergenic
Count
TATA
ATAT
ATAT
TATA
CGGC
5-mers
ATATA
TATAT
CCGGC
GCGGC
CGGCC
6-mers
ATATAT
TATATA
CCGGCC
GCGGCC
CCCCGG
Log2 ratio (nucleosome/genome)
Enrichment in nucleosomes

Figure S14. Binding preferences of nucleosomes located in genes and intergenic regions. Nucleosome enrichment of 4-mers, 5-mers and 6-mers that are exclusively composed of A/T or G/C nucleotides is shown. For both gene regions (top rows) and intergenic regions (bottom rows), enrichment is expressed as the log 2 ratio of the frequency of a DNA-mer inside nucleosomes and the genome-wide frequency of that DNA-mer. Enrichment of 5-mers at the ring stage is shown in the main manuscript (Fig. 4C-D).

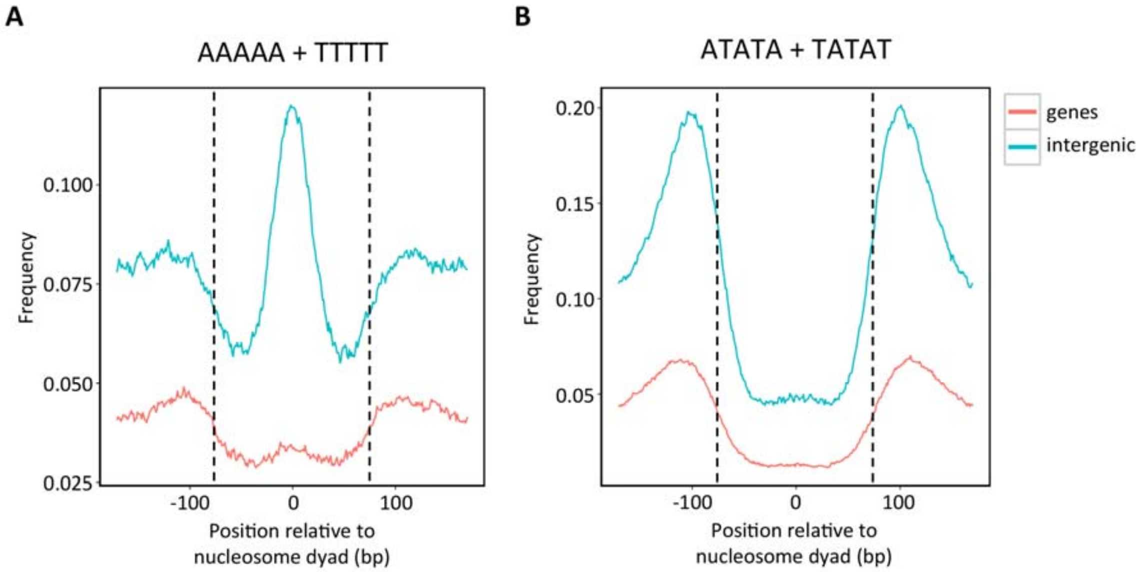

Figure S15. Distribution of nucleosome-disfavoring sequences in *P. falciparum* nucleosomes. A. Frequency of poly(dA:dT)$_5$ tracts in nucleosomes. Poly(dA:dT) tracts are strongly disfavorable for nucleosome binding in other eukaryotes, but show an increased frequency at the dyad in nucleosomes located in intergenic regions of the *P. falciparum* genome. **B**. Frequency of AT-repeat 5-mers in nucleosomes. AT-repeats are strongly disfavored for nucleosome binding in the *P. falciparum* genome. Dashed lines indicate nucleosome boundaries.

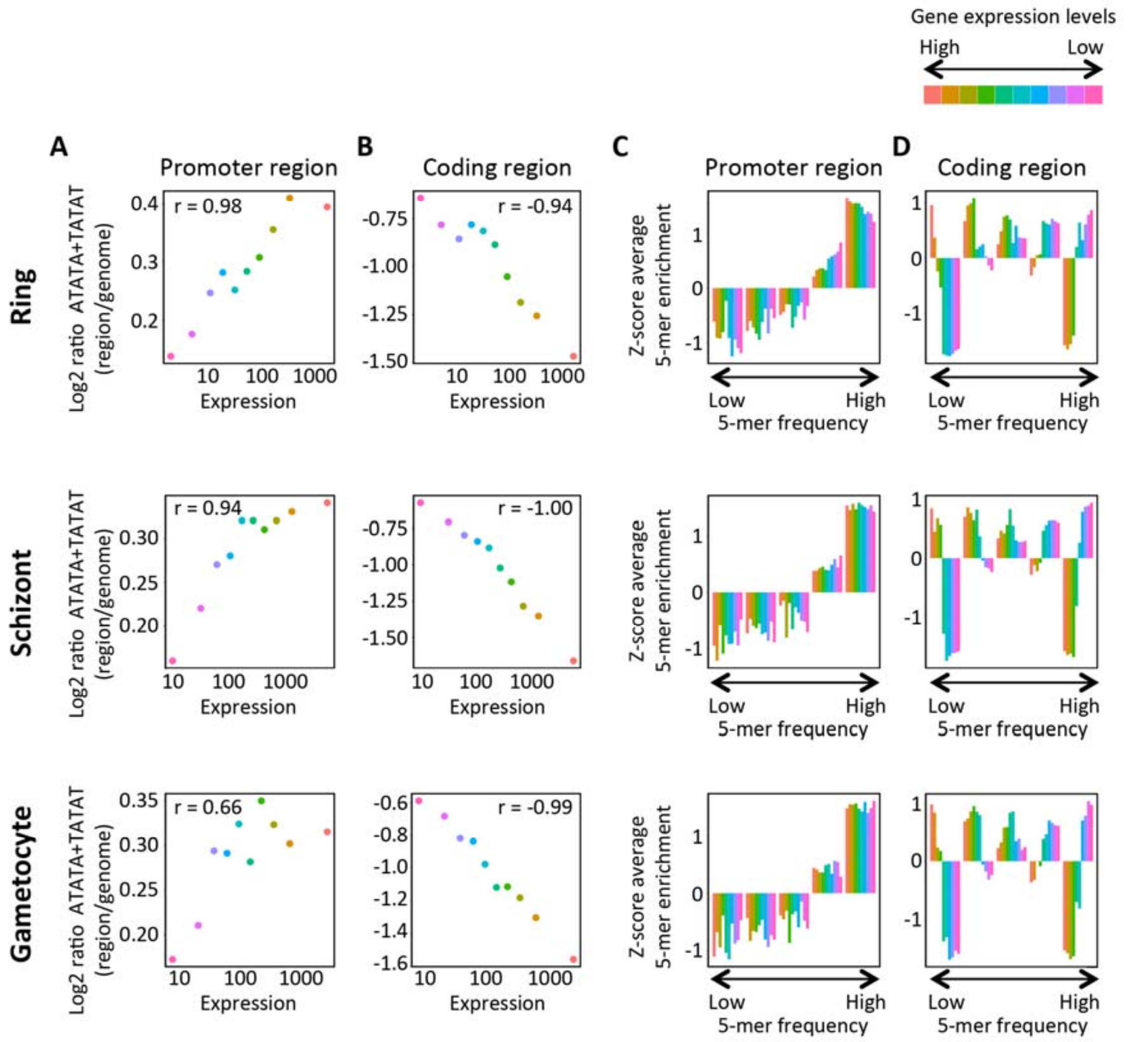

Figure S16. Association between sequence composition and transcription levels. A-B. Enrichment of AT-repeat 5-mers (ATATA and TATAT) in 500 nucleotides upstream of the translation start site (promoter region; A) and coding region (B) relative to their genome-wide frequency, for genes in each transcription cluster at ring (top row), schizont (middle row) and gametocyte (bottom row) stages. Correlation coefficients were obtained using the Spearman's rank test. **C-D**. Sequence composition in promoter regions (C) and coding regions (D) among genes with different expression levels at ring (top row), schizont (middle row) and gametocyte (bottom row) stages. 5-mers were divided into five groups based on their genome-wide frequency in intergenic regions. For each transcription cluster, the z-normalized average log 2 ratio of 5-mer frequency in the gene or promoter region versus all genes or intergenic regions, respectively, is plotted for all 5-mer frequency groups. Highly expressed genes show an increased prevalence of nucleosome-favorable 5-mers, while genes with low transcription levels show an increased prevalence of nucleosome-disfavorable 5-mers.

Table S1. Overview of sequence reads mapped to the human and *P. falciparum* genomes.

			Sequence reads per stage ($\times 10^6$)			
Sample	Ab	Organism	Ring	Troph.	Schizont	Gameto.
Sonication ChIP-Seq	Non-specific	*P. falciparum*	0.2	0.2	0.2	n.a.
Sonication ChIP-Seq	H3	*P. falciparum*	4.5	2.5	7.2	n.a.
		Human	21.7	17.6	19.2	n.a.
MNase Chip-Seq	H3	*P. falciparum*	20.1	4.0	17.1	n.a.
		Human	1.6	4.6	1.4	n.a.
MAINE-Seq (This study)	n.a.	*P. falciparum*	n.a.	n.a.	n.a.	35.4
		Human	n.a.	n.a.	n.a.	18.2
MAINE-Seq (Bartfai *et al.*) (20)	n.a.	*P. falciparum*	4.0	8.5	6.2	n.a.
		Human	0.9	5.1	0.2	n.a.

Ab: ChIP antibody; troph.: trophozoite; gameto.: gametocyte; n.a.: not applicable.

Table S2. Normalization factors

Stage	Ratio Pf:human DNA	% Pf sequence reads[a]			Normalization	
		MNase ChIP-Seq	Sonication ChIP-Seq	MAINE -Seq	Ratio[b]	Factor[c]
Ring	1:1	92.7[d]	17.2	82.3[f]	0.927	1.000
Troph.	2.5:1	46.4	12.4	62.7[f]	0.464	0.500
Schizont	10:1	92.3	27.3	96.7[f]	0.923	0.995
Gameto.	1:1 – 6:1[e]	n.a.	n.a.	66.0	0.660	0.712

Pf: *P. falciparum*; troph.: trophozoite; gameto.: gametocyte; n.a.: not applicable.

[a] Percentage of sequence reads mapped to the *P. falciparum* genome out of all sequence reads mapped to either the human or the *P. falciparum* genomes.

[b] Normalization ratios were calculated by dividing the number of reads mapped to the *P. falciparum* genome by the total number of reads mapped to both human and *P. falciparum* genomes. Ratios of the MNase ChIP-Seq experiment are shown for asexual parasite stages.

[c] Final normalization factor was obtained by scaling the normalization ratio relative to the ring stage.

[d] Ring stage parasites for the MNase ChIP-Seq experiment were obtained early in the next erythrocytic cycle, after re-invasion of parasites (6 hours after the schizont stage), resulting in a relatively low fraction of human DNA contamination.

[e] Ratio of *P. falciparum* to human DNA in gametocyte cultures are likely to differ from those in asexual stage cultures, as a result of lower parasitemia and decreasing numbers of viable human white blood cells after two weeks of culture.

[f] MAINE-Seq data for the asexual stages was derived from a previously published nucleosome occupancy data set (20).
